# Loss of BAF Complex in Developing Cortex Perturbs Radial Neuronal Migration in a WNT Signaling-Dependent Manner

**DOI:** 10.3389/fnmol.2021.687581

**Published:** 2021-06-16

**Authors:** Godwin Sokpor, Cemil Kerimoglu, Huong Nguyen, Linh Pham, Joachim Rosenbusch, Robin Wagener, Huu Phuc Nguyen, Andre Fischer, Jochen F. Staiger, Tran Tuoc

**Affiliations:** ^1^Institute for Neuroanatomy, University Medical Center Goettingen, Göttingen, Germany; ^2^Department of Human Genetics, Ruhr University of Bochum, Bochum, Germany; ^3^German Center for Neurodegenerative Diseases, Göttingen, Germany; ^4^Faculty of Biotechnology, Thai Nguyen University of Sciences, Thai Nguyen, Vietnam; ^5^Department of Neurology, University Medical Center Heidelberg, Heidelberg, Germany; ^6^Neurooncology Clinical Cooperation Unit, German Cancer Research Center, Heidelberg, Germany; ^7^Department for Psychiatry and Psychotherapy, University Medical Center Göttingen, Göttingen, Germany; ^8^Cluster of Excellence “Multiscale Bioimaging: from Molecular Machines to Networks of Excitable Cells” (MBExC), University of Göttingen, Göttingen, Germany

**Keywords:** BAF complex, neuronal migration, cortical lamination, glial fibers, cell adhesion, Wnt signaling, cortical development

## Abstract

Radial neuronal migration is a key neurodevelopmental event indispensable for proper cortical laminar organization. Cortical neurons mainly use glial fiber guides, cell adhesion dynamics, and cytoskeletal remodeling, among other discrete processes, to radially trek from their birthplace to final layer positions. Dysregulated radial migration can engender cortical mis-lamination, leading to neurodevelopmental disorders. Epigenetic factors, including chromatin remodelers have emerged as formidable regulators of corticogenesis. Notably, the chromatin remodeler BAF complex has been shown to regulate several aspects of cortical histogenesis. Nonetheless, our understanding of how BAF complex regulates neuronal migration is limited. Here, we report that BAF complex is required for neuron migration during cortical development. Ablation of BAF complex in the developing mouse cortex caused alteration in the cortical gene expression program, leading to loss of radial migration-related factors critical for proper cortical layer formation. Of note, BAF complex inactivation in cortex caused defective neuronal polarization resulting in diminished multipolar-to-bipolar transition and eventual disruption of radial migration of cortical neurons. The abnormal radial migration and cortical mis-lamination can be partly rescued by downregulating WNT signaling hyperactivity in the BAF complex mutant cortex. By implication, the BAF complex modulates WNT signaling to establish the gene expression program required for glial fiber-dependent neuronal migration, and cortical lamination. Overall, BAF complex has been identified to be crucial for cortical morphogenesis through instructing multiple aspects of radial neuronal migration in a WNT signaling-dependent manner.

## Introduction

Neuronal migration can be considered as a patterning event which affords gross and subtle anatomical and functional cortical area establishment during brain development (Silva et al., 2019). For the most part, neuronal migration is the critical process that ensures proper placement of groups of neurons into their fated cortical laminae during morphogenesis of the cortex. Hence, in the event of neuronal misplacement due to abnormal migration, the cortex is mis-laminated and the ectopic neurons become susceptible to developmental anomalies, including abnormal differentiation, incorrect neurite formation and synaptogenesis, and dysregulated cell death, which can culminate in many neurodevelopmental disorders (Valiente and Marin, 2010; Evsyukova et al., 2013; Severino et al., 2020).

The bona fide cortical excitatory neurons generated by radial glia (RG) cells in the ventricular zone (VZ) or by neurogenic progenitors in the subventricular zone (SVZ) of the developing mouse cortex make challenging radial navigations from their place of birth to reside in defined regions (layers) in the cortical plate (CP) by means of radial migration (Noctor et al., 2001, 2002; Pontious et al., 2008). Radial migration can occur in the form of somal translocation or locomotion (Nadarajah et al., 2001). During somal translocation, which is mainly used by early born neurons, the nascent neuron elaborates a long leading process anchored at the pial basement membrane. By means of progressive traction force generated by shortening of the long leading process, the soma of the neuron is continually translocated to be placed in its designated cortical lamina (Miyata et al., 2001; Nadarajah et al., 2001).

Locomotion on the other hand is a complex and multiphasic process. Unlike somal translocation, it depends on the molecular and structural guidance of RG fibers needed for radial profiles of neuronal movement that largely contribute to the formation of superficial neocortical layers (Rakic, 1972). The locomoting neuron, mainly late-born, displays striking morphological changes in the course of its trajectory. Notably, during locomotion, the newborn neuron briefly attaches to its mother glial fiber or the adjoining fiber and actively moves with a bipolar morphology into the lower part of the intermediate zone (IZ). In the IZ, the bipolar neuron disengages from the glial fiber to momentarily pause radial migration. It then transitions to or adopts a multipolar morphology with which it makes undefined micro-movements to probably collect directional cues for subsequent radial (oriented) migration. Upon adequate molecular conditioning and transient NMDA receptor-mediated glutamatergic synaptic stimulation, the multipolar neuron then switches back to bipolar morphology in the vicinity of the upper IZ and re-attaches to the glial fiber to resume locomotion to its final destination in the CP (Kriegstein and Noctor, 2004; Noctor et al., 2004; Inoue et al., 2014; Mizutani, 2018; Ohtaka-Maruyama et al., 2018). The bipolar neuron characteristically extends a pia-directed leading process, the dendrite-to-be, and a trailing process toward the VZ which becomes the future axon (Rakic et al., 1996). Adopting the appropriate neuronal morphology or polarity is a key determinate of successful radial migration and cortical layer formation, which when perturbed can lead to cortical malformation (Hakanen et al., 2019). Locomotion ends in the CP by detachment of the migrating neuron from the glial fiber to be properly positioned in its home layer via somal translocation (Nadarajah et al., 2001).

Indeed, radial neuronal migration is a complex cell biological process which must be under tight molecular regulation. As such, a myriad of factors, including transcriptional and signaling factors, have been identified to spatiotemporally regulate various aspects of cortical neuron radial migration (reviewed in Heng et al., 2007; Marin et al., 2010; Evsyukova et al., 2013). Notably, it has been shown in seminal studies that the formation and maintenance of RG fibers, and related neuronal cell adhesion dynamics are tightly regulated during radial migration (Anton et al., 1997, 1999; Elias et al., 2007; Kawauchi et al., 2010; Shikanai et al., 2011; Sild and Ruthazer, 2011; Valiente et al., 2011; Solecki, 2012; Desai et al., 2013; Evsyukova et al., 2013; Schmid et al., 2014; Tonosaki et al., 2014; Jinnou et al., 2018; Louhivuori et al., 2018; Schaffer et al., 2018; Zhang et al., 2019).

Epigenetic factors have lately been at the center stage of neurodevelopment regulation after previous underestimation of their phenomenal role in orchestrating neural development. Emerging among these epigenetic regulators are the chromatin remodelers which can redesign the epigenetic landscape to influence gene expression and related cell biological events through direct alteration of chromatin structure and/or the recruitment of other epigenetic or transcriptional cofactors during cortical development (Sokpor et al., 2018).

The Brg1/Brm-associated factor (BAF) complex, a mammalian version of the yeast SWI/SNF complex, is a multi-subunit protein complex which primarily functions as a chromatin remodeler (Clapier et al., 2017) and has been shown in recent years to be indispensable for neural development (Son and Crabtree, 2014; Sokpor et al., 2017, 2018). During cortical development, the BAF complex regulates key processes such as specification, proliferation, differentiation, and functional maturation of cortical progenitors or postmitotic neurons (Son and Crabtree, 2014; Sokpor et al., 2017, 2018). Notably, the BAF complex subunits can be reconstituted to form cell type specific variants that have unique functional effects. For example, there are some compositional and functional differences between the BAF complex in neural progenitors (npBAF) and the BAF complex in neurons (nBAF) (Lessard et al., 2007; Wu et al., 2007; Kadoch et al., 2013; Tuoc et al., 2013; Bachmann et al., 2016).

Although chromatin remodelers, including some BAF subunits, have been reported to regulate neuronal migration in the developing mammalian cortex (Nott et al., 2013; Wiegreffe et al., 2015; Nitarska et al., 2016; Xu et al., 2018) and in worm neural tissue (Weinberg et al., 2013), the mechanism involved is far from clear. In this current study, we aimed at elucidating the molecular and cellular mechanisms by which the BAF complex orchestrates migration of excitatory projection neurons in the developing cortex. To this end, we abolished the BAF complex in early and late cortical progenitors and specifically in postmitotic neurons to investigation how BAF complex(es) influence neuronal migration during corticogenesis. From our molecular and cellular analyses of the BAF complex mutant (knockout and knockdown) cortex, it was evident that neurons fail to migrate properly in the absence of BAF complex functionality. As a result, cortical neurons are misplaced in the mutant cortex leading to abnormal cortical cytoarchitectonic and concomitant laminar malformation. The BAF complex-ablated cortical neuron is incapable of proper radial migration because of loss of glial fiber guides and cell adhesion, defective cell polarization, and abnormal Wingless/Int (WNT) signaling activity. Indeed, the said altered intrinsic and extrinsic elements are known to be vital for correct radial migration, and are tightly modulated by many regulatory factors during cortical development (reviewed in Evsyukova et al., 2013); to which we here add BAF complex as a critical component.

## Materials and Methods

### Animal Handling and Generation of Transgenic Mice

We applied guidelines of the German Animal Protection Law in handling the animals. Floxed BAF155 (Choi et al., 2012), floxed BAF170 (Tuoc et al., 2013), Emx1-Cre (Gorski et al., 2002), hGFAP-Cre (Zhuo et al., 2001), and Nex-Cre (Goebbels et al., 2006) transgenic mice were used in the study. All animals were maintained in a C57BL6/J background.

To eliminate BAF155 and BAF170 in early or late cortical progenitors, and in postmitotic neurons, we crossed mice carrying floxed BAF155 and BAF170 genes with the early progenitor-active Emx1-Cre (Gorski et al., 2002) or late progenitor-active hGFAP-Cre (Zhuo et al., 2001) and neuron-specific Nex-Cre (Goebbels et al., 2006) mouse lines to generate dcKO_Emx1-Cre, dcKO_hGFAP-Cre, and dcKO_Nex-Cre mutants, respectively. Heterozygous animals (BAF155fl/+, BAF170fl/+, Cre negative) were used as controls. Emx1-Cre mutants die before birth, whereas hGFAP-Cre and Nex-Cre mutants survive early postnatal stages.

### Plasmids

The following plasmids were used in the study: pCIG2-eGFP, pCIG2-Cre-ires-eGFP (gift from Prof. Francois Guillemot, NIMR London; Hand et al., 2005), and NeuroD-Cre-ires-GFP, NeuroD-GFP (gift from Prof. Laurent Nguyen, University of Liège, CHU Sart Tilman, Liège, Belgium).

### Antibodies

Commercially obtained monoclonal (mAb) and polyclonal (pAb) primary antibodies used in the study: CTIP2 rat pAb (1:200; Cat. ab18465; Abcam), Cux1 rabbit pAb (1:50; Cat. sc-13024, Santa Cruz), GM130 rat mAb (1:100; Cat. 610823; BD), BAF170 rabbit pAb (1:100; Cat. HPA021213; Sigma), BAF60a mouse mAb (1:200; Cat. 611728; BD), BAF155 rabbit pAb (1:20; Cat. sc-10756; Santa Cruz), BRM rabbit pAb (1:200; Cat. ab15597; Abcam), BRG1 rabbit pAb (1:120; Cat. sc-10768X; Santa Cruz), BAF155 mouse mAb (1:100; Cat. sc-48350X; Santa Cruz), BAF250b mouse mAb (1:100; Cat. WH0057492M1; Sigma), Tbr1 rabbit pAb (1:300; Cat. ab31940; Abcam), α-Catenin rabbit pAb (1:200; Cat. C2081; Sigma), Pax6 mouse mAb (1:100; Developmental Studies Hybridoma Bank), Nestin mouse mAb (1:50; Cat. 611658, BD), Pax6 rabbit pAb (1:200; Cat. PRB-278P; Covance), GFP chicken pAb (1:500; Cat. ab13970; Abcam). Secondary antibodies used were Alexa 488-, Alexa 568-, Alexa 594- and Alexa 647-conjugated IgG (various species, 1:400; Molecular Probes).

### RNA Sequencing

RNA sequencing (RNA-seq) and analyses were performed as previously described in Narayanan et al. (2015, 2018), Nguyen et al. (2018). The high throughput RNA-seq data has been deposited in the NCBI Gene Expression Omnibus and accessible via the accession number GSE106711 and also at Narayanan et al. (2015), Nguyen et al. (2018).

### Immunohistology and *in situ* Hybridization

Immunohistochemical staining and *in situ* hybridization of cortical tissue sections were performed as previously described (Tuoc et al., 2013; Bachmann et al., 2016; Wagener et al., 2016). The following RNA probes were used in the *in situ* hybridization experiment: *Ndnf* (*A930038C07Rik*), *Rorb* (*Rorbeta*), *Etv1* (*Er81*), and *TC1460681* (simply designated *TC*), to label cortical layers 1, 4, 5, and 6, respectively (Wagener et al., 2016).

### Imaging and Quantitative Analysis

Coronal mouse brain sections were imaged with confocal (TCS SP5, Leica) and/or widefield fluorescence (Axio Imager M2, Zeiss; fitted with Neurolucida software, MBF Bioscience) microscopes. Further image processing was done with Adobe Photoshop program.

Neuronal cell counting and distribution (bin analysis), and leading process length estimation were performed using NIH ImageJ software. Neuronal cells with nuclear or cytoplasmic labeling for specific markers with or without DAPI staining, were counted in 4–6 structurally-matched control and mutant (dcKO) or electroporated cortical sections obtained from 3 to 4 biological replicates. Fluorescent signal intensity measurement was used to quantify uncountable histological staining in confocal images using ImageJ software as previously reported (Tuoc and Stoykova, 2008; Narayanan et al., 2015).

### *In utero* Electroporation

*In utero* electroporation was done as previously described (Tabata and Nakajima, 2001; Tuoc and Stoykova, 2008; Tuoc et al., 2013). In brief, the pregnant mouse was surgically operated to expose the E14.5 embryos in the intact uterus. About 3 μL of a mixture of the plasmid of interest (2 μg/μL) and 0.5% fast green, at a ratio of 1:10, was then injected into one lateral ventricle of the embryo’s brain. Transfection of the cortical neuroepithelium was achieved by applying 5 pulses of current (∼30 V) across the brain, with the positive terminal of the electroporator on the injected side of the cortical hemisphere. Every other embryo was injected and electroporated for each set of embryos in the uterus. Embryos were then returned into the abdominal cavity and the surgical incision was closed. The brains of the electroporated embryos were then harvested at E17.5 for histological processing and microscopic analysis.

### Pharmacological Treatment Using WNT Inhibitor

ICG001 (Tocris Bioscience, Cat. No. 4505), was dissolved in DMSO (vehicle). Pregnant mice received daily intraperitoneal injections of vehicle (150 μL), or ICG001 (150 μL of a 1.0 mg/mL solution) from 11.5 to 16.5 days post coitum (d.p.c.). The brains of the treated embryos (control and mutant) were harvested and processed for histological analysis at Embryonic day (E) 17.5.

### Statistical Analyses

Prism was used to perform statistical analyses. Statistical comparisons were carried out using Student’s *t* test or its non-parametric equivalent, the Mann–Whitney *U* Test, and one-way ANOVA followed by Bonferroni’s multiple comparison (*Post Hoc*) test or the non-parametric equivalent Kruskal–Wallis test followed by Dunn’s multiple comparison test, where appropriate. The results are presented as means ± SEM or median and range for non-parametric data.

## Results

### Loss of BAF Complex in Cortical Progenitors Results in Impaired Neuronal Migration Leading to Cortical Mis-Lamination

We previously identified that the BAF complex function is abolished by double deletion of its scaffolding subunits BAF155 and BAF170. In the absence of BAF155 and BAF170, the entire BAF complex stability is compromised. This leads to disassembly of other components (subunits) of the complex, making them susceptible to proteasomal degradation with attendant functional inactivation of the entire BAF complex (Narayanan et al., 2015; Bachmann et al., 2016; Nguyen et al., 2016, 2018).

**FIGURE 1:**
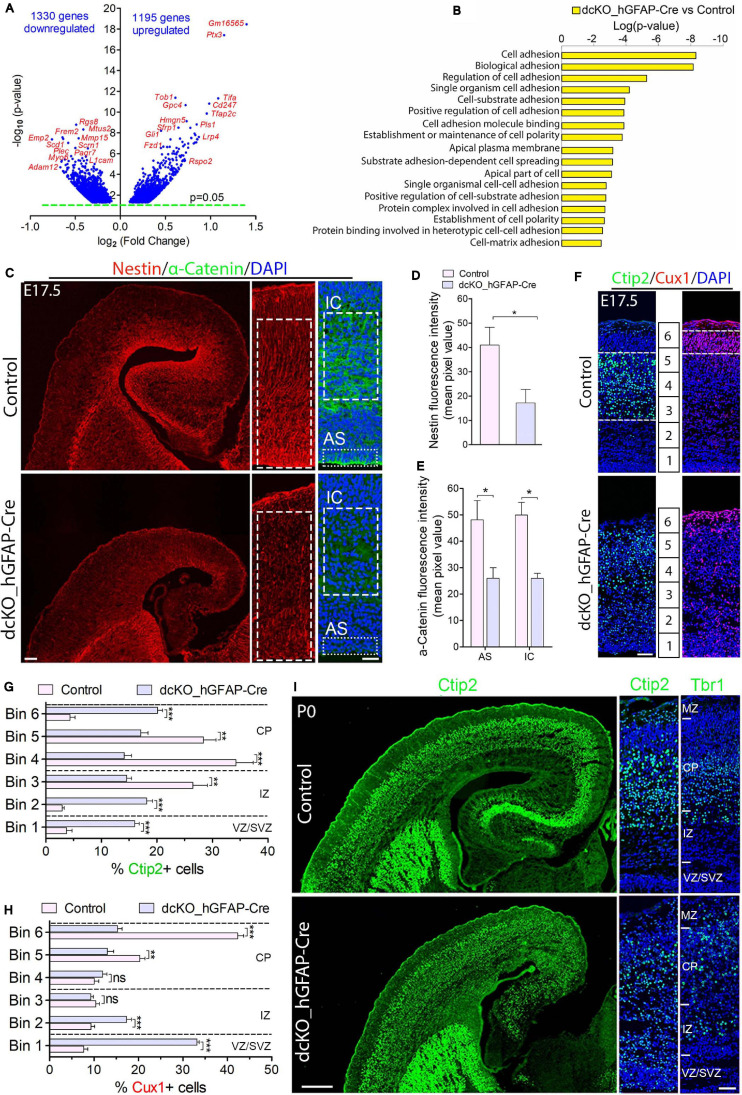
Cortical layers are malformed in the absence of BAF complex, and attributable to loss of cell adhesion and glial fiber scaffolds. **(A)** Volcano plot showing genes downregulated and upregulated in the E17.5 dcKO_hGFAP-Cre cortex. Examples of top altered genes are indicated. **(B)** Graph showing downregulation of selected gene categories or pathways mainly related to cell adhesion and polarity formation in the E17.5 dcKO_hGFAP-Cre cortex. **(C)** Sections of E17.5 control and dcKO_hGFAP-Cre cortex immunostained for the glial fiber protein Nestin and the cell adhesion protein α-Catenin. Specifically quantified cortical areas are shown with rectangles with dashed or stippled lines. **(D,E)** Simple **(D)** and grouped **(E)** bar charts showing quantification of Nestin and α-Catenin, respectively, in the E17.5 control and dcKO_hGFAP-Cre cortex. **(F)** Micrographs showing immunostaining with antibodies against Ctip2 and Cux1 to mark neurons that make the lower (deep) and upper (superficial) cortical layers, respectively, in the E17.5 control and dcKO_hGFAP-Cre cortex. White dashed lines are used to delineate the deep (Ctip2+) and superficial (Cux1+) cortical layers. Bins (1–6) for neuronal distribution analysis are indicated. **(G,H)** Bar charts showing quantitative distributions of Ctip2+ deep layer neurons and Cux1+ superficial layer neurons in the E17.5 control and dcKO_hGFAP-Cre cortical wall. Quantified cortical area = (420 μm × 170 μm). **(I)** Micrographs showing the P0 control and dcKO_hGFAP-Cre cortex with Ctip2 and Tbr1 immunostaining. Where shown, sections are counterstained with DAPI (blue). Unpaired Student’s *t*-test was used to test for statistical significance: *p < 0.01, **p < 0.001, ***p < 0.0001; ns, not significant; *n* = 6. Scale bars: = 100 μm and 50 μm in overview and zoomed images, respectively. Results are presented as mean ± SEM. IC, intracortical; AS, apical surface; VZ, ventricular zone; SVZ, subventricular zone; IZ, intermediate zone; CP, cortical plate; MZ, marginal zone.

Given that cortical mass is dramatically reduced when BAF complex is abolished in the neuroepithelium of early developing cortex at the onset of neurogenesis (Narayanan et al., 2015), we were unable to comprehensively study neuronal migration in the mouse cortex that has lost BAF155 and BAF170 from E10.5 onward as achieved in the dcKO_Emx1-Cre cortical model (Supplementary Figures 1, 2). This warranted our choice of another mutagenic strategy that allowed us to delete the BAF complex at a later stage of corticogenesis. Thus, we generated the dcKO_hGFAP-Cre mouse forebrain model in which BAF155 and BAF170 are deleted predominately in the VZ progenitors of the developing cortex to achieve ablation of the entire BAF complex (Supplementary Figure 1; Nguyen et al., 2018). Because the hGFAP-Cre is relatively late-acting, with the earliest activity detected around E13.5 (Zhuo et al., 2001; Nguyen et al., 2018), we were able to lessen the impact of loss of BAF complex on cortical morphogenesis compared with that caused in the dcKO_Emx1-Cre (Figure 1 vs. Supplementary Figure 2). Therefore, the dcKO_hGFAP-Cre model allowed us to study neuronal migration at late embryonic and early postnatal stages of cortical development with fairly preserved cortical integrity in the absence of BAF complex.

We characterized the neuronal migration phenotype of the E17.5 cortex, by which time the knockout effect under the hGFAP-Cre activity is fully established in the entire cortex (Nguyen et al., 2018). We started by reanalyzing our previously generated RNA-seq data from the E17.5 cortex in which 1329 genes and 1195 genes were downregulated and upregulated, respectively (Figure 1A; Nguyen et al., 2018). We then screened for gene categories implicated in the regulation of neuron migration in the cortex. Consistent with our observations in the E12.5 dcKO_Emx1-Cre cortex (Supplementary Figures 2A,B), we found several gene pathways involved in neuronal cell migration, cell polarity establishment, neurite formation, and cell adhesion downregulated in the dcKO_hGFAP-Cre cortex (Figure 1B). Given that these categories of factors play crucial roles in oriented-neuronal migration (locomotion) to afford proper cortical lamination, we further probed the dcKO_hGFAP-Cre cortex for specific factors that can affect the radial migration of neurons therein. We selectively focused on the integrity of radial glial scaffolds and cell adhesion in the BAF complex mutant cortex due to their striking roles in radial migration (Nadarajah et al., 2001; Kriegstein and Noctor, 2004; Noctor et al., 2004; Drees et al., 2005; Elias et al., 2007; Schmid et al., 2014; Schaffer et al., 2018). Thus, immunohistochemical investigations for the adhesion protein α-Catenin and the Nestin+ RG fibers in the E17.5 control and dcKO_hGFAP-Cre cortex, which are notably downregulated in the dcKO_Emx1-Cre cortex (Supplementary Figures 2C–E), were performed. As expected, we found demonstrable depletion of RG fibers and reduction in the apical and intra-cortical expression of cell adhesion (α-Catenin) in the dcKO_hGFAP-Cre cortex as compared with control (Figures 1C–E). Alternative proteins that indicate radial glial fiber identity (BLBP, GLAST, RC2) and related cell adhesion proteins (ZO1, Occludin) were shown to be reduced in the BAF complex-ablated developing cortex (Narayanan et al., 2015; Nguyen et al., 2018). Moreover, the delamination, dispersion, and hyperproliferation of BAF155 and BAF170-deficient neural stem cells (Pax6-expressing cortical progenitors) also reflect loss of their fiber-mediated anchorage in the cortical wall (Narayanan et al., 2015, 2018; Nguyen et al., 2018; Xie et al., 2019). These observations imply that radial glial scaffolds are actually lost in the absence of BAF complex functionality.

The neuronal migration phenotype in the E17.5 dcKO_hGFAP-Cre and control developing cortex was assessed by applying antibodies against the lower layer (L5) cortical neuron marker protein Ctip2 (Arlotta et al., 2005; Gaspard et al., 2008) and the cortical layer (L2/3) neuron marker protein Cux1 (Nieto et al., 2004). It was revealed that loss of BAF complex at later-stage of embryonic corticogenesis severely disturbed migration of both lower (L5) and upper layer (L2/3) neurons in the E17.5 dcKO_hGFAP-Cre. Neurons expressing Ctip2 or Cux1 were observed to spread in the entire E17.5 dcKO_hGFAP-Cre cortex instead of forming their respective layers as outlined in the control images (Figures 1F–H). Because the dcKO_hGFAP-Cre mutants barely survive early postnatal life, we limited the postnatal characterization of the migration phenotype to P0 (i.e., just after birth), and by which time the majority of lower layer cortical neurons [Tbr1 + (L6) and Ctip2 + (L5) neurons] have largely completed somal translocation or locomotion to properly settle in their respective layers (Nadarajah et al., 2001; Marin and Rubenstein, 2003; Molyneaux et al., 2007). Unlike Cux1 or Brn2+ neurons, Tbr1+ or Ctip2+ neuron production is minimally affected by hGFAP-Cre-mediated BAF155 and BAF170 deletion in the early postnatal cortex (Nguyen et al., 2018). Therefore, we immunohistologically compared the distribution of lower layer neurons in the P0 control and dcKO_hGFAP-Cre cortex (Figure 1I). Quantitative analysis indicated significant mis-distribution of Ctip2+ L5 neurons in the P0 dcKO_hGFAP-Cre cortex as compared with control (Supplementary Figure 3A and Figure 1I). Although, majority of the Tbr1 + L6 neurons migrated out of the germinal zone, they appear to have over-migrated, making them locate in the upper layer domain in the P0 dcKO_hGFAP-Cre cortex as compared with control (Supplementary Figure 3B and Figure 1I). Additional check also showed that Brn2-expressing upper layer neurons are also unable to migrate properly in the dcKO_hGFAP-Cre cortex, leading to their accumulation in the lower half of the E18.5 mutant cortical wall instead of settling in their destined upper cortical layers as in control (Supplementary Figures 3C,D).

Together, these results strongly implicate the role of the BAF complex in orchestrating radial migration of cortical neurons and proper cortical layer development. However, just like in the dcKO_Emx1-Cre model (Supplementary Figure S2F), the migration phenotype in the dcKO_hGFAP-Cre cortex co-existed with disruption in progenitor cell proliferation and differentiation consequent to BAF complex inactivation in neural stem cells (Narayanan et al., 2015; Nguyen et al., 2018). Thus, a postmitotic neuron-specific disruption of BAF complex function is necessary to exclude abnormal neurogenesis in our cortical neuron migration model.

### Ablation of BAF Complex in Postmitotic Neurons Caused Abnormal Migration of Upper Cortical Layer Neurons

Specifically targeting nascent cortical neurons for BAF complex inactivation is necessary to study migration defects independent of key extrinsic factors such as glial fibers or cell adhesion elements essential for radial migration. To achieve the neuron-specific ablation of BAF complex, we resorted to the Nex-Cre line (Goebbels et al., 2006). We generated the dcKO_Nex-Cre mouse line in which BAF155 and BAF170 are deleted exclusively in the principal neurons generated in the cortex from E10.5 onward (Supplementary Figure 1; Goebbels et al., 2006). The dcKO_Nex-Cre cortex can be seen to have lost the expression of other core subunits of the BAF complex together with BAF155 and BAF 170, although some residuals can be seen in the mutant cortex, which are likely expressed by glia or interneurons that are not affected by the NeuroD-Cre activity (Supplementary Figure 4).

**FIGURE 2:**
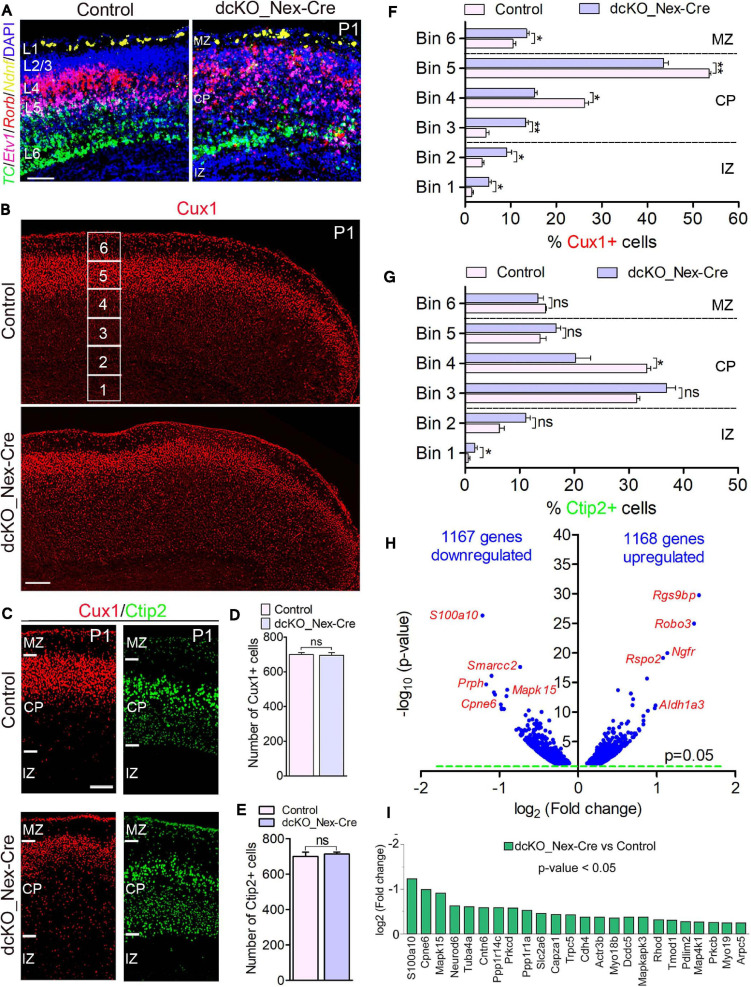
Neuron-specific ablation of BAF complex causes downregulation of cell morphogenesis-related genes leading to delayed neuronal migration. **(A)** Micrographs showing fluorescence *in situ* hybridization in the P1 control and dcKO_Nex-Cre cortex stained with the layer-specific RNA probes *TC1460681*, *Etv1*, *Rorb*, and *Ndnf* to reveal cortical layers 6, 5, 4, and 1, respectively. **(B)** Overview micrographs showing immunostaining of the P1 control and dcKO_Nex-Cre cortex with Cux1 antibody to mainly mark cortical layer 2/3 neurons. Bins (1–6) for neuronal distribution analysis are indicated. **(C)** Micrographs with cortical regions indicated showing Cux1 and Ctip2 immunostaining of the P1 control and dcKO_Nex-Cre cortex. **(D,E)** Bar charts showing comparable number of Cux1+ **(C)** and Ctip2+ **(D)** neurons in the P1 control and dcKO_Nex-Cre cortical wall. **(F,G)** Bar graphs showing quantitative distribution (Bin analysis) of Cux1+ **(F)** and Ctip2+ **(G)** neurons in the P1 control and dcKO_Nex-Cre cortical wall. Quantified cortical area = (720 μm × 400 μm). **(H)** Volcano plot show genes downregulated and upregulated in the P1 dcKO_Nex-Cre cortex compared with control. Examples of top altered genes are shown. **(I)** Bar chart showing specific neuronal morphogenesis-related genes with reduced expression in the P1 dcKO_Nex-Cre cortex. Where shown, sections are counterstained with DAPI (blue). Unpaired Student’s *t*-test was used to test for statistical significance: *p < 0.01, **p < 0.001, ns, not significant; *n* = 6. Scale bar = 100 μm. Results are presented as mean ± SEM. IZ, intermediate zone; CP, cortical plate; MZ, marginal zone.

Characterization of the migration phenotype in the dcKO_Nex-Cre cortex was mainly done at early postnatal stage P1 due to early lethality of mutants. Generally, the gross forebrain phenotype of the dcKO_Nex-Cre cortex was less severe as compared with the dcKO_hGFAP-Cre cortex (data not shown). To identify subtle migration defects, we first performed fluorescence *in situ* hybridization (FISH) experiment in which the P1 dcKO_Nex-Cre and control cortex were riboprobed with the layer specific markers *TC*, *Etv1*, *Rorb*, and *Ndnf* which label the cortical layers 6, 5, 4, 1, respectively. Based on distribution of the aforementioned FISH probe signals, it was observed that cortical layers are much less defined in the CP of the P1 dcKO_Nex-Cre cortex compared with control (Figure 2A). The pattern particularly gives an impression of a wide spreading of upper layer neurons in the CP, with lower layer neurons displaying a mild form of such amorphous distribution in the P1 dcKO_Nex-Cre cortex (Figure 2A). Because the Ndnf-marked L1 neurons are Cajal-Retzius cell which migrate tangentially into the cortex from other cortical areas like the hem (Bielle et al., 2005), they are presumably not affected by the Nex-Cre-mediated ablation of BAF complex; hence they displayed no disturbance in their laminar fate in the P1 mutant cortex compared to control (Figure 2A).

At the protein level, our immunohistochemical analysis showed that loss of BAF complex led to a mild defect in the migration of Cux1+ upper layer neurons in the P1 dcKO_Nex-Cre cortex compared with control (Figures 2B,C), without significantly affecting the generation of such superficial cortical layer neurons (Figure 2D). Analysis using Brn2 immunolabeling corroborated the abnormal (mildly delayed) migration of upper layer neurons lacking BAF155 and BAF170 (Supplementary Figure 5). However, migration and distribution of Ctip2+ lower layer neurons, which are also normally generated in the dcKO_Nex-Cre cortex, were less obviously affected (Figures 2C,E). Indeed, bin analyses revealed fewer number of Cux1+ neurons reached Bins 4 and 5 in the dcKO_Nex-Cre cortex compared with control (Figure 2F), whereas the distribution of Ctip2+ neurons is arguably fairly normal in the absence of BAF155 and BAF170 (Figure 2G). Of note, unlike in the dcKO_hGFAP-Cre cortex (Figures 1C,D), radial glial fiber density or layout seems not to be disturbed in the dcKO_Nex-Cre cortex (Supplementary Figures 6A–D) following the deletion of BAF155 and BAF170 in postmitotic neurons. Therefore, the upper layer neuron migration defect observed is likely independent of problems with the radial glial scaffolds in the dcKO_Nex-Cre cortex.

Consistent with the observed phenotype, we identified pertinent abnormal alterations in gene expression program in the P1 dcKO_Nex-Cre cortex that emphasized or may have underlined the disturbed neuronal migration. We found in our RNA-seq analysis that almost equal number of genes are downregulated (1167) as upregulated (1168) in the P1 dcKO_Nex-Cre cortex compared with control (Figure 2H). Notably, some of the top downregulated genes include those that are associated with neuronal migration. No overt change in the expression of cell adhesion genes was observed (Figure 2H, see RNA-seq data sheet). Interestingly, we noticed that many of the neuronal migration-related genes downregulated in neurons lacking BAF complex are key for cytoskeletal remodeling (Figure 2I), and needed for cell polarization and morphogenesis critical for cell migration (de la Torre-Ubieta and Bonni, 2011). Thus, the neurons in the P1 dcKO_Nex-Cre cortex may be incapable of adopting the right morphology suitable for their radial migration.

Altogether, we have shown that exclusive deletion of BAF complex in postmitotic neurons perturbs the expression of genes crucial for neuronal morphogenesis. This can lead to improper formation of neuronal appendages necessary for oriented-neuronal migration, and which has implication for defective or sluggish locomotion of cortical neurons.

### Knockdown of BAF155 and BAF170 in Cortical Progenitors Led to Disoriented Neuronal Migration in Developing Cortex

**FIGURE 3:**
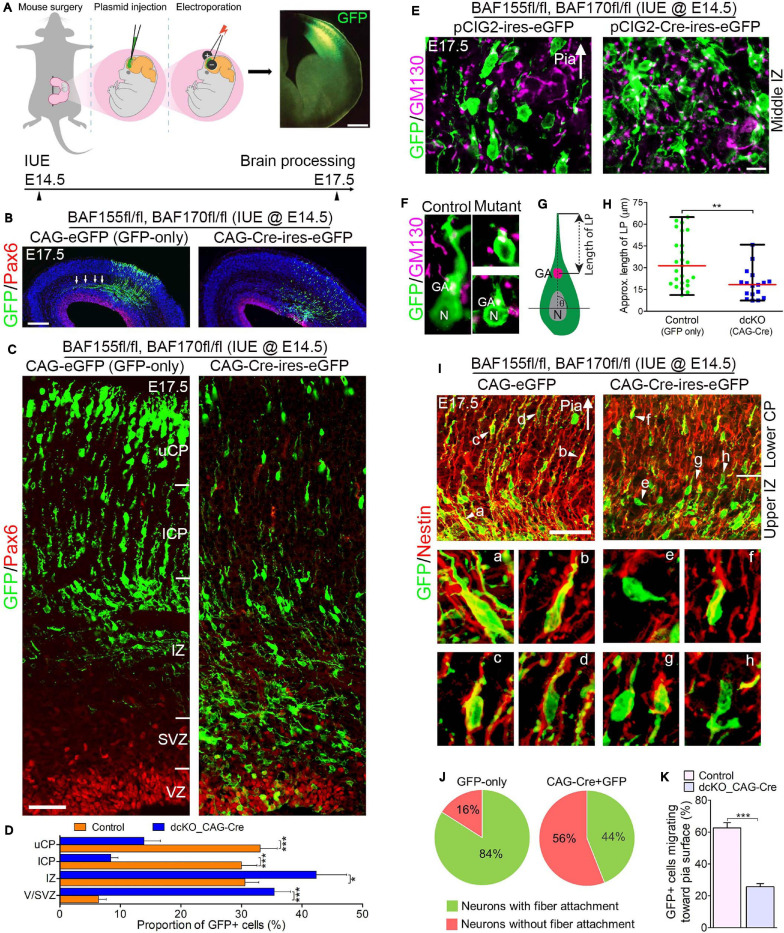
Locomoting neurons without BAF complex exhibit abnormal polarity and radial direction mis-guidance. **(A)** Illustration of *in utero* electroporation technique used to achieve knockdown of BAF155 and BAF170. It shows a representative embryo in the uterine horn bearing double floxed BAF155 and BAF170 (BAF155fl/fl, BAF170fl/fl), and being injected in the brain with the plasmid of interest and electroporated at E14.5. Brain tissue was harvested at E17.5. A representative immunomicrograph showing GFP expression in E15.5 cortex is presented. **(B)** Immunohistochemical micrographs showing overview of Pax6 (to mark the germinal zone) and GFP staining in the E17.5 cortex (BAF155fl/fl, BAF170fl/fl) electroporated with control (GFP-only) or CAG-Cre + GFP plasmids. Arrows in control image point to outgrowth of axons. **(C)** Micrographs showing the electroporated cortical areas in **(B)** at higher magnification. Approximate cortical wall regions are shown. **(D)** Bar chart showing distribution of GFP+ cells (neurons) in the various cortical regions in the control and CAG-Cre + eGFP plasmid-injected E17.5 cortex. **(E)** Images showing the E17.5 control and mutant cortical middle intermediate zone with GFP and GM130 staining (Golgi apparatus marker). Arrow points in the direction of the pia surface. **(F–H)** Micrographs with GFP and GM130 staining **(F)** and illustration **(G)** showing estimation of the leading process (LP) length of control and BAF complex mutant cortical neurons in the lower cortical plate, which is graphically (statistically) compared in **(H)**. **(I)** Images of the E17.5 control and mutant upper intermediate zone and lower cortical plate immunostained with GFP and Nestin antibodies to reveal neuron-glial fiber colocalization. Arrow heads (a–h) indicate examples of neurons migration with (a–d) or without/abnormally with (e–h) glial fiber guide. Arrow points in the direction of the pia surface. **(J)** Pie chart depicting the quantification of the proportion of GFP+ control and BAF complex mutant neurons migrating with or without glial fiber guide in the upper intermediate zone and lower cortical plate. **(K)** Bar chart showing the proportion of GFP+ neurons with their Golgi-leading process axis oriented toward the pia in **(I)**. Arrows in **(E,I)** points in the direction of the pia surface. Where shown, sections are counterstained with DAPI (blue). Unpaired Student’s *t*-test was used to test for statistical significance: *p < 0.01, ***p < 0.0001; *n* = 4, selected in total 25 and 17 control and mutant GFP+ neurons in the upper IZ/lower CP region for leading process length estimation, respectively. Scale bars: =200 and 50 μm in overview and zoomed images, respectively. Results are presented as mean ± SEM or median and range. IUE, *in utero* electroporation; GA, Golgi apparatus; N, nucleus; VZ, ventricular zone; SVZ, subventricular zone; IZ, intermediate zone; l/uCP, lower/upper cortical plate; MZ, marginal zone.

In order to refine the cortical neuron migration phenotype due to loss of BAF complex so as to identify any alteration in the cellular dynamics involved, we employed the *in utero* electroporation technique (Figure 3A). This focal genetic ablation strategy afforded sparse loss of BAF complex in selected/single cortical neurons, thus making the resultant migration phenotype more conspicuous. To achieve this, we electroporated the mouse cortex double floxed for BAF155 and BAF170 with control plasmid pCIG2-eGFP (CAG-eGFP/GFP-only) or effector plasmid pCIG2-Cre-ires-eGFP (CAG-Cre-eGFP) into the E14.5 cortex (Figure 3A). Because the CAG-Cre is active in the cortical neuroepithelium, we essentially mimicked the neuronal migration phenotype due to loss of BAF complex under the Emx1- or hGFAP-Cre promoters as observed in the dcKO_Emx1-Cre and dcKO_hGFAP-Cre cortical models reported earlier (Supplementary Figure 2 and Figure 1). The electroporated embryos were allowed to develop until E17.5 and then cortical tissue was collected for immunohistological analyses.

By means of GFP immunostaining, we were able to track the progress of transfected migrating cortical neurons from the Pax6-marked VZ to the CP. We observed formation and fasciculation of the axons of the GFP-only transfected neurons, whereas the CAG_Cre-GFP transfected neurons failed to show noticeable axon formation; signifying possible differentiation defect in the neurons lacking BAF155 and BAF170 (Figure 3B). This can be linked to abnormal RG cell fiber-neuron contact or adhesion, which can disrupt axon formation and orientation during locomotion (Xu et al., 2015).

At higher magnification, it was evident that glial fiber-dependent (radial) migration of cortical neurons was disrupted following loss of BAF155 and BAF170. Compared with control, the BAF complex mutant (i.e., CAG_Cre-GFP-treated) neurons, presumably multipolar neurons, appeared to have accumulated in the lower regions of the cortical wall namely the Pax6-labeled VZ and SVZ, and the IZ, making the upper and lower aspects of the CP less populated with successfully migrated and/or properly migrating neurons (Figures 3C,D). We identified that the cortical neurons without functional BAF complex were mis-oriented with respect to their normal radial alignment and also exhibited abnormal polarity. This was revealed in our immunohistological examination of the Golgi apparatus localization and leading process length of migrating neurons in the middle and upper IZ (Figures 3E,F). The BAF complex-ablated bipolar neuron in the upper IZ had its GM130+ Golgi located close to the nucleus and presented a reduced leading process length (*p*-value = 0.0032, Mann–Whitney *U* Test; Figures 3F–H).

In further support of our assertion of defective directed-neuronal migration when BAF complex is inactivated in neurons, we observed reduced engagement of Nestin + glial fibers by the CAG_Cre-GFP-transfected migrating neurons compared with the GFP-only positive neurons in the upper IZ and lower CP (Figure 3I). In other words, based on neuron-glial fiber proximity (i.e., GFP and Nestin signal “colocalization”), whereas more than 80% of the control migrating neurons depend on or use the glial fiber scaffolds to radially migrate, far less proportion (16%) of CAG_Cre-GFP positive neurons seem to use glial fibers for migration—even in the presence of normal radial profiles of glial fibers (Figures 3I,J). Moreover, given the role of adhesion molecules in radial migration (Solecki, 2012), we inferred from the loss of cell adhesion in the developing cortex due to deletion of BAF155 and BAF170 (Supplementary Figure 1 and Figures 1A–E) that it is possible the CAG_Cre-GFP positive neurons improperly attach to glial fibers (or not at all) because of loss of glial fibers and/or adhesion proteins leading to abnormal radial migration. Indeed, the decreased proportion of migrating neurons facing the pia, in mutants (CAG_Cre-GFP-transfected neurons) compared with control (GFP-only-transfected neurons) (Figure 3K) gives reason to abnormal radial migration seen in the BAF complex knockdown condition (Figures 3B,C).

In all, the reduced use of glial fibers for migration by the BAF complex-ablated cortical neurons and their defective Golgi-dependent polarization, may have contributed to their aberrant radial orientation and migration of neurons in the developing cortex.

### BAF155 and BAF170-Deficient Migrating Cortical Neurons Display Defective Multipolar-to-Bipolar Transition

Applying the same logic and advantage of using the dcKO_Nex-Cre line as opposed to the dcKO_Emx1-Cre and hGFAP-Cre lines, we neuron-specifically knocked-down BAF155 and BAF170 in the E14.5 cortex using a NeuroD-Cre-ires-GFP plasmid in another *in utero* electroporation experiment as in Figure 3A. Thus, the NeuroD-Cre-mediated inactivation of BAF complex in postmitotic nascent cortical neurons increased the specificity of the phenotypic effect on radially migrating neuron as compared with the CAG-Cre approach, which targeted the BAF complex in neural stem or progenitor cells.

**FIGURE 4:**
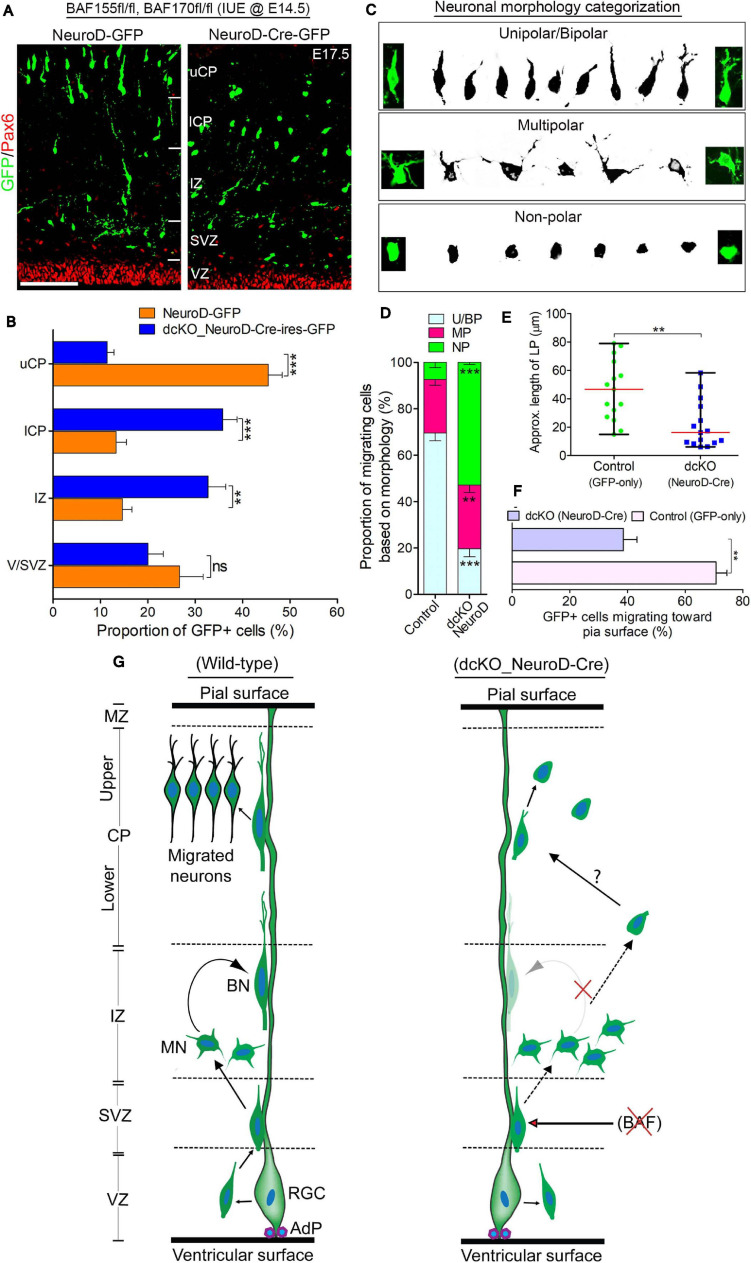
BAF complex regulates multipolar-to-bipolar neuronal morphology transition during radial migration. **(A)** Micrographs showing Pax6 and GFP immunostaining in the E17.5 cortex (BAF155fl/fl, BAF170fl/fl) electroporated with control (NeuroD-GFP) or Cre (NeuroD-Cre-ires-GFP) plasmids. Approximate cortical regions are shown. **(B)** Bar chart showing percentage distribution of GFP+ neurons in the various cortical regions in the control (NeuroD-GFP) and NeuroD-Cre-ires-GFP plasmid-injected E17.5 cortex. **(C)** Images showing representative morphological groupings of the migrating neurons with or without BAF155/BAF170 (BAF complex) in the E17.5 cortex. **(D)** Composite bar graph showing the quantitative proportions of the various categories of neuronal morphologies (in **C**) in the E17.5 cortical areas electroporated with control (NeuroD-GFP) or NeuroD-Cre-ires-GFP plasmids. **(E)** Graph comparing the estimated leading process length of migrating neurons transfected with control or NeuroD-Cre plasmids. **(F)** Bar graph comparing the proportion of neurons migrating toward the pia surface in the cortex electroporated with control and NeuroD-Cre plasmids. **(G)** Graphical summary of the abnormal neuronal migration due to BAF complex ablation in neurons as compared with control. Solid (curved) arrows indicate normal transition, broken straight arrows denote abnormal morphology transition, and red crossed lines indicate suppression. Unpaired Student’s *t*-test was used to test for statistical significance: **p < 0.001, ***p < 0.0001; ns, not significant; *n* = 4. Results are presented as mean ± SEM or median and range. Scale bar: =50 μm. IUE, *in utero* electroporation; VZ, ventricular zone; SVZ, subventricular zone; IZ, intermediate zone; l/uCP, lower/upper cortical plate; MZ, marginal zone; U/BN, unipolar/bipolar neuron; MN, multipolar neuron; RGC, radial glial cell; AdP, adhesion protein.

Examination of GFP immunostaining in E17.5 cortex, double floxed for BAF155/BAF170, electroporated with control (NeuroD-GFP) and NeuroD-Cre-ires-GFP plasmids at E14.5 revealed marked disruption of radial neuronal migration in the absence of BAF complex (Figure 4A). While the Pax6-stained VZ in the cortex transfected with NeuroD-GFP or NeuroD-Cre-ires-GFP plasmids is devoid of GFP+ cells, the IZ and lower CP of the NeuroD-Cre-ires-GFP electroporated cortex are filled with more GFP+ cells compared with the control cortex (Figures 4A,B). However, as an indication of successful radial migration, the control upper CP is seen to be populated with significantly more GFP+ cells as compared with the NeuroD-Cre-ires-GFP treated cortex (Figures 4A,B).

One of the critical cellular processes during radial migration of cortical neurons is the transient transformation from multipolar to bipolar morphology, which occurs in the upper IZ (Nadarajah et al., 2001; Noctor et al., 2004). The function of the multipolar phase is not clearly known, albeit some believe directional cues are collected by the many temporary neurites of the neurons at this stage (Mizutani, 2018; Shikanai et al., 2018). The bipolar structure on the other hand is suited for the glial fiber attachment and subsequent active locomotion to the CP (Nadarajah et al., 2001; Noctor et al., 2004). This led us to in examining the morphological integrity of migrating neurons lacking BAF complex. We sampled and grouped the diverse forms of migrating neurons in the electroporated regions of the cortical wall into three morphological categories: unipolar/bipolar, multipolar, and non-polar (neurite-lacking) neurons (Figure 4C). Our statistical quantification revealed a significantly smaller proportion of bipolar or unipolar neurons in the NeuroD-Cre-ires-GFP electroporated cortical area compared with the NeuroD-GFP electroporated cortical area. However, we found more multipolar neurons in the NeuroD-Cre-ires-GFP electroporated cortex compared with the cortical area electroporated with NeuroD-GFP plasmid (Figure 4D). Strikingly, we also found more so-called non-polar neurons in the NeuroD-Cre-ires-GFP treated cortex, particularly in the CP, as compared with that in the control cortex. Such non-polar neurons presented only with their soma (Figures 4A,C,D). Consistent with observations in the CAG_Cre-GFP IUE experiment, neurons transfected with NeuroD-Cre-ires-GFP plasmid display short leading process (Figure 4E), and many of the leading processes were also not directed toward the pia surface (Figure 4F).

**FIGURE 5:**
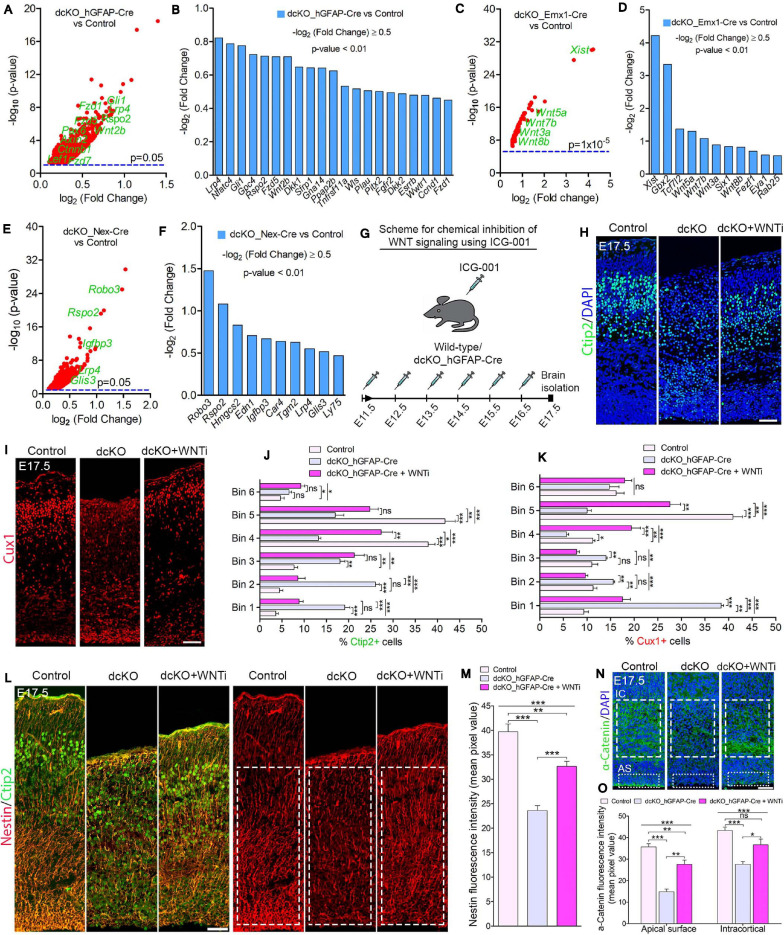
BAF complex modulates WNT signaling to drive neuronal migration and cortical lamination. **(A–F)** Volcano plots **(A,C,E)** and bar charts **(B,D,F)** showing upregulation of WNT signaling-related or target genes in the E17.5 dcKO_hGFAP-Cre **(A,B)**, E12.5 dcKO_Emx1-Cre **(C,D)**, and P1 dcKO_Nex-Cre cortex **(E,F)**. **(G)** Illustration of the scheduled intraperitoneal injection, of pregnant mouse carrying dcKO_hGFAP-Cre embryos, with the WNT inhibitor (WNTi) ICG001. **(H,I)** Immunomicrographs showing Ctip2 **(H)** and Cux1 **(I)** – labeled neurons in the E17.5 control, dcKO_hGFAP-Cre, and WNT inhibitor-treated dcKO_hGFAP-Cre cortex. **(J,K)** Bar graphs showing quantitative bin analyses to compare the distribution of Ctip2+ **(J)** and Cux1+ **(K)** neurons in the E17.5 control, dcKO_hGFAP-Cre, and WNTi-treated dcKO_hGFAP-Cre cortex. **(L)** Immunomicrographs showing Nestin and Ctip2 (merged), and Nestin-only staining in the E17.5 control, dcKO_hGFAP-Cre, and WNT inhibitor -treated dcKO_hGFAP-Cre cortex. **(M)** Bar chart indicating quantification of Nestin+ glial fibers partial rescued in the WNT inhibitor-treated dcKO_hGFAP-Cre cortex compared with the dcKO_hGFAP-Cre and control cortex. **(N)** Images showing intracortical (IC) and apical surface (AS) expression of α-Catenin in the E17.5 control, dcKO_hGFAP-Cre, and WNT inhibitor-treated dcKO_hGFAP-Cre cortex. **(O)** Graphical representation of the quantification of α-Catenin expression following WNT inhibition in the dcKO_hGFAP-Cre cortex compared with that in the dcKO_hGFAP-Cre and control cortex. Inserted rectangles with dashed or stippled lines indicate specific cortical areas quantified. One-way ANOVA followed by Bonferroni *post hoc* analysis was used to test for significance: *p < 0.05, **p < 0.005, ***p < 0.0005; ns, not significant; *n* = 4–6. Scale bar: = 50 μm. Results are presented as mean ± SEM.

Based on these observations and as schematized in Figure 4G, our findings implicate the BAF complex in the regulation of multipolar-to-bipolar transition of cortical neurons during radial migration. As such, in the event of BAF complex dysfunction (i.e., under NeuroD-Cre-ires-GFP treatment condition), this neuronal morphology conversion is hindered, leading to accumulation of multipolar neurons in the IZ at the expenses of bipolar neurons in the CP (Figures 5A,D,G). Moreover, it is likely that even if it occurs, the morphological transformation of the BAF complex-lacking neurons is abnormal and results in aberrantly polar neurons with truncated and mis-oriented leading processes (Figures 4A,E,F). Thus, it is conceivable that the BAF complex mutant neurons undergo unconventional or complicated migration other than the suitable radial (glial-guided) migration that is prerequisite for correct laminar formation in the developing cortex.

Together, we identified the BAF complex to also regulate the process of multipolar-to-bipolar neuronal morphology transition, which is a rate-determining phase in the process of radial migration permissive for correct establishment of the various cortical layers–especially upper cortical layers–during development of the mouse cortex.

### BAF Complex May Repress WNT Signaling to Permit Proper Neuronal Migration and Cortical Lamination During Brain Development

In order to identify possible molecular factors or mechanism that mediate BAF complex influence on radial migration of cortical neurons, we screened the results of our RNA-seq data generated for the BAF complex mutant and control developing cortex. With reference to our previous report that BAF complex regulates cortical neurogenesis via suppression of WNT signaling in the developing mouse cortex (Nguyen et al., 2018), we asked whether the neuronal migration phenotype in the BAF complex mutant brain is dependent on WNT signaling. Interestingly, several signaling pathways, including WNT signaling, have been implicated in neuronal migration regulation during brain development (Siegenthaler and Miller, 2004; Hashimoto-Torii et al., 2008; Yi et al., 2010; Boitard et al., 2015; Bocchi et al., 2017; Martinez-Chavez et al., 2018; Saxena et al., 2018). In the case of WNT signaling, it was reported that dynamic regulation of the cascade is necessary for multipolar-to-bipolar morphology transition during radial migration of cortical neurons (Boitard et al., 2015).

**FIGURE 6:**
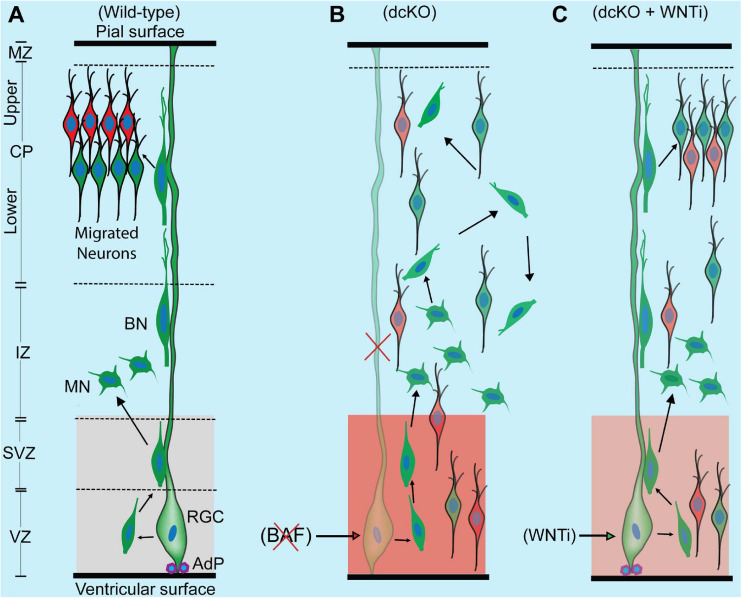
Schematic synopsis of how BAF complex may drive radial neuronal migration. **(A)** Schema showing the normal course of radial neuronal migration in the developing wild-type cortex. The parent cortical neural progenitor that generates majority of excitatory neurons is the radial glia cell (RGC). It is typically anchored at the ventricular surface by adhesion proteins (AdP) and extends a long slender fiber that traverses the marginal zone (MZ) to be anchored at the pial surface. The fiber acts as scaffold that supports the radially migrating neurons. After the RGC gives rise to a newborn neuron in the ventricular zone (VZ), the young postmitotic neuron usually attaches to the parent glial fiber and quickly migrates into the border between the subventricular (SVZ) and intermediate zones (IZ), where it acquires a multipolar neuronal (MN) identity and briefly stops migrating to receive spatiotemporal molecular cues for further oriented migration. The MN then switches to bipolar neuron (BP) and re-attaches to the glial fiber to migrate into the cortical plate (CP). In the CP, the migrating neuron detaches from the glial fiber to undergo somal translocation leading to its correct layer placement and ensures proper cortical lamination. The optimal activity of WNT signaling in the cortical germinal zone is indicated by a light shade of red and critical for neuronal migration. **(B)** Schema showing that loss of BAF complex in neural progenitors causes loss of adhesion proteins, glial fiber, disturbance of MN-to-BN transition, and increase in WNT signaling activity (indicated with a deep shade of red). These alterations result in accumulation of nascent neurons and MNs, leading to abnormal radial migration and cortical laminar malformation. Red cross denotes deletion or loss. **(C)** Picture showing outcome of rescue experiment following inhibition of the increased WNT signaling in the BAF complex mutant (dcKO_hGFAP-Cre) cortex. WNT inhibition (WNTi) with the chemical ICG001 led to substantial preservation of neuronal polarization, glial fibers, and cell adhesion leading to partial rescue of radial neuronal migration and cortical lamination.

Gene set enrichment analyses indicated abnormal elevation of WNT signaling activity in the BAF complex mutant cortex. Thus, WNT signaling-related or target genes were significantly upregulated in the BAF complex mutant cortex (Figures 5A–F). Notably, the E17.5 dcKO_hGFAP-Cre displayed more WNT signaling-related factors with increased expression (Figures 6A,B; Nguyen et al., 2018) compared with that observed in the E12.5 dcKO_Emx1-Cre (Figures 5C,D) or P1 dcKO_Nex-Cre cortex (Figures 5E,F). Therefore, we considered the WNT signaling pathway as a potential candidate for rescuing the neuronal migration phenotype. To this end, we designed a rescue experimental paradigm for reducing the WNT signaling hyperactivity in the mutant cortex. We chose the dcKO_hGFAP-Cre cortex for our rescue experiment. The reason being that the dcKO_Emx1-Cre cortex is not appropriate for clearly visualizing neuronal migration dynamics because of massive cortical atrophy (Supplementary Figure 2F), and the observation that the migration phenotype in the dcKO_Nex-Cre cortex is mild and most likely to be more of a delayed rather than stalled neuronal migration (Figures 2A,B and Supplementary Figure 5).

To perform the rescue experiment, we employed the *in vivo* chemical inhibition approach to downregulate (knockdown) the upregulated WNT signaling in the developing dcKO_hGFAP-Cre cortex. This was achieved with the chemical ICG-001, a small molecule capable of interfering with CREB-binding protein (CBP) and β-catenin complexing during WNT signaling, leading to inhibition of the pathway and its related downstream molecular and cellular effects (Emami et al., 2004; Teo et al., 2005; Wiese et al., 2017). Mice pregnant with dcKO_hGFAP-Cre embryos were thus intraperitoneally injected with daily dose of ICG-001 from E11.5 to E16.5 and the brains were harvested for histological analysis at E17.5 (Figure 5G). We previously established that ICG-001 treatment at the dosage applied in this study, or treatment with the vehicle (DMSO), does not affect neurogenesis or neuronal migration in the wildtype cortex. Similarly, treatment with DMSO had no effect on cortical neurogenesis or neuronal migration in the BAF mutant brain (Nguyen et al., 2018).

Intriguingly, immunostaining with Ctip2 and Cux1 antibodies revealed partial rescue of radial neuronal migration and formation of deep and superficial cortical layers in the WNT inhibitor (WNTi)-treated dcKO_hGFAP-Cre cortex compared with the dcKO_hGFAP-Cre without WNTi pharmacological treatment (Figures 5H,I). Our bin analysis showed that the apparent crowding of Ctip2+ lower layer neurons and Cux1+ upper layer neurons in the E17.5 dcKO_hGFAP-Cre lower cortical wall region significantly resolved due to migration of many of such neurons to populate their designated laminae in the CP upon downregulation of WNT signaling (Figures 5J,K). We also observed more preserved RG fibers and α-Catenin (cell adhesion) expression in the WNTi-treated dcKO_hGFAP-Cre cortex compared with dcKO_hGFAP-Cre (Figures 5L–O). In agreement with the migration rescue effect of WNT inhibition in the dcKO_hGFAP-Cre, we observed that more WNT inhibitor-treated mutant (dcKO_hGFAP-Cre) neurons labeled with GFP migrated out of the germinal zone and the IZ to populate the upper CP compared with neurons in the WNT inhibitor-untreated dcKO_hGFAP-Cre cortex (Supplementary Figures 7A,B). Furthermore, we observed slight increase in the leading process of the rescued (WNT inhibitor-treated) BAF complex mutant neurons compared with the untreated mutant neurons (Supplementary Figure 7C). It is probable that WNT inhibition in the dcKO_hGFAP-Cre cortex afforded maintenance of glial fibers and cell adhesion, and rescue of the defective multipolar-to-bipolar neuronal morphology transition, which culminated in a largely correct radial neuronal migration and cortical layer formation (Figure 6).

Put together, we have shown that the BAF complex is a critical regulator of the glial fiber-dependent and independent aspects of radial migration of cortical neurons through modulation of WNT signaling activity to allow proper cortical lamination during mouse brain development.

## Discussion

Laminar patterning during cortical development is fundamental to the formation of functional cortical areas during development of the cerebral cortex. Neuronal migration is a critical process in cortical layer formation, and its dysregulation can render the cortex malformed with several resultant neurodevelopmental disorders (Valiente and Marin, 2010; Evsyukova et al., 2013; Silva et al., 2019). Therefore, factors that regulate migration of cortical neurons have been of interest to many neurobiologists over the years.

In this study, we identified the ATP-dependent chromatin remodeling BAF complex to play instructional roles during radial migration of cortical neurons. We essentially inactivated the BAF complex in neurogenic cortical progenitors or in nascent postmitotic cortical neurons prior to commencement of radial migration. This was carried out using three different mouse models, in two of which the BAF complex was inactivated in cortical progenitors at early and later cortical development stages to produce the dcKO_Emx1-Cre and dcKO_hGFAP-Cre, respectively. Abrogation of the BAF complex just after generation of postmitotic cortical neurons was achieved in the dcKO_Nex-Cre model. Thus, the dcKO_Nex-Cre model offered the advantage of precluding the impact of abnormal proliferation and neurogenesis seen/reported in the dcKO_Emx1-Cre and dcKO_hGFAP-Cre cortical models (Narayanan et al., 2015; Nguyen et al., 2018). We observed that the BAF complex-deficient cortex lacks well defined cortical layers due to neuronal migration dysregulation. In principle, given that the composition and functional characteristics of the BAF complex in neural progenitors (npBAF) is different from that found in neurons (nBAF) (Lessard et al., 2007; Wu et al., 2007; Staahl et al., 2013; Bachmann et al., 2016), to a large extent, we were able to dissect the significance of BAF complexes in orchestrating neuronal migration during cortical histogenesis.

### BAF Complex Orchestrates Cortical Lamination via Regulating Neuron Migration in the Developing Cortex

By temporally and cell-type specifically inactivating the BAF complex in the developing cortex, we found that the resultant mutant neurons are incapable of normal migration. As a result, the BAF complex-ablated developing cortex is improperly laminated; showing both upper and lower layer neuron misplacement in the cortical wall. The defective neuronal migration phenotype was most severe and morphogenically impactful in the dcKO_Emx1-Cre cortex followed by the dcKO_hGFAP-Cre cortex. This may be as a result of concurrent disturbance of neurogenesis due to deletion of BAF complex in the early or late cortical neural stem/progenitor cells. Indeed, neural progenitor cell proliferation and differentiation are perturbed in the absence of optimal BAF complex function (Narayanan et al., 2015; Nguyen et al., 2018). On the other hand, the migration phenotype in the dcKO_Nex-Cre cortex was mild and mainly presented as delayed locomotion of upper layer neurons because they are late-born and make the longest radial journey. This reduced impact in the dcKO_Nex-Cre cortex may have resulted from the relative stability of the BAF complex in postmitotic neurons probably because of their non-dividing nature and the non-requirement of subunit recomposition of the nBAF therein (Lessard et al., 2007; Wu et al., 2007). As such, although majority of the BAF subunits are lost, we found some subunit remnants in the P1 dcKO_Nex-Cre cortex (Supplementary Figure 3), which we speculate may underlie the observed mild phenotype.

Typically, abnormal neuronal migration calls forth several other neurodevelopmental perturbations (Valiente and Marin, 2010; Evsyukova et al., 2013). In our case, we found that the defective cortical neuronal migration may, in part, underscore the defective morphology of the BAF complex mutant neurons, which contributed to the diminished corticogenesis in the BAF complex mutant brain (Narayanan et al., 2015; Nguyen et al., 2016, 2018). At least, in terms of differentiation, some BAF complex subunits have been reported to be essential for axonogenesis, dendritogenesis, and spine formation during cortical neuron maturation (reviewed in Sokpor et al., 2017).

Another reason that consolidates the specific link of BAF complex dysfunction to the observed neuronal migration problem is that: it is only under the condition of entire BAF complex ablation that we disturb cortical neuron migration. Single deletion of BAF155 or BAF170 did not yield any noticeable neuronal migration anomaly in the developing cortex (Tuoc et al., 2013; Narayanan et al., 2018). That notwithstanding, a previous study reported defective neuronal migration following deletion of Ctip1 (BAF100a), a variable subunit of the BAF complex (Wiegreffe et al., 2015). We argue, however, that BAF100a may act solitarily outside the chromatin remodeling function of the BAF complex to control aspects of cortical neuron migration. Even though the defective migration caused by deletion of BAF155 and BAF170 is more complex and severe than that resulting from BAF100a ablation alone, it would be insightful to investigate the structural and functional integrity of the BAF100a-lacking BAF complex and how it may contribute to defective neuronal migration during brain development.

### Essential Cellular Mechanics in Radial Neuronal Migration Require BAF Complex Function

Classically, *in utero* electroporation is a powerful *in vivo* technique used for gene manipulation to investigate cellular processes, including neuronal migration in the developing cortex (Saito and Nakatsuji, 2001; Tabata and Nakajima, 2001; Shimogori and Ogawa, 2008). We took advantage of this method to focally ablate BAF complex in the developing cortex, so as to detail the effect on cellular mechanisms during neuronal migration. To closely reproduce loss of BAF complex in cortical progenitors as achieved in the dcKO_Emx1-Cre and dcKO_hFGAP-Cre cortex, and in postmitotic neurons as in the dcKO_Nex-Cre cortex, we used the CAG- and NeuroD-Cre plasmids to delete BAF155 and BAF170 in selected cortical areas, respectively. By this means, we identified that key steps involved in glial fiber-guided cortical neuron migration go awry in the absence of BAF complex. Notably, we found that most of the BAF complex-deficient migrating neurons “could not” locomote properly possibly because of a defect in attachment to glial fibers. The observed reduction in neuronal-glial fiber interaction may partly be due to disturbance in cell adhesion to available glial fibers as revealed in our RNA-seq analyses of the dcKO_Emx1-Cre and dcKO_hFGAP-Cre cortex. Adhesion molecules such as catenins and cadherins, play central roles in the attachment of neurons to glial guides during locomotion (Solecki, 2012). In agreement, it has been recently shown that loss of adhesion proteins in the cortex, particularly α-Catenin, can perturb radial neuronal migration leading to cortical neurodevelopmental disturbances (Schmid et al., 2014; Schaffer et al., 2018). This partly gives relevance to the link between loss of α-Catenin in the BAF complex-inactivated cortex and contribution to the defective radial migration, and cortical mis-lamination phenotype reported in this study.

Although not obviously revealed in our BAF complex knockdown investigation using *in utero* electroporation, it is conceivable that the reduced association of the BAF complex-lacking migrating cortical neurons with glial fibers may also emanate from the loss of the glial fiber layouts needed for radial migration. This is deduced from the dcKO_Emx1-Cre and dcKO_hFGAP-Cre cortex, which display dramatic loss of glial fiber scaffolds due to ablation of BAF complex (Narayanan et al., 2015; Nguyen et al., 2018). Traditionally, nascent cortical neurons use their parent glial fibers to locomote to the CP to make well-patterned radial columns, cortical laminae, and functional areas defined in the cortex (Mountcastle, 1997; Noctor et al., 2001; Torii et al., 2009). Neuronal migration may stall or deviate in the absence of such proximal glial fiber guidance, which accounts for the accumulation of Ctip2+ and Cux1+ neurons in the lower cortical wall of the dcKO_hGFAP-Cre cortex and in the germinal zones of the cortical area electroporated with the CAG-Cre-GFP plasmid. Moreover, our observation indicates that the migrating BAF complex-ablated neurons may display protracted multipolar phase leading to their accumulation in the IZ and/or may undergo excessive tangential migration in attempt to find adjoining fibers for onward radial migration (Inoue et al., 2017).

Adding to the complexity of how BAF complex regulates cortical neuron migration, we also identified its importance in controlling the multipolar-to-bipolar neuronal morphology switch during radial migration. This morphological transformation is critical for successful radial migration (Noctor et al., 2004; Ayala et al., 2007; Namba et al., 2014) and its interference or dysregulation can stifle neuronal migration (La Fata et al., 2014; Boitard et al., 2015; Chen et al., 2015; Barnat et al., 2017; Guo et al., 2017; Huang et al., 2017; Iwai et al., 2018; Kurabayashi et al., 2018; Ohtaka-Maruyama et al., 2018; Zhang et al., 2018). The BAF complex-ablated cortical neurons are unable to properly undergo multipolar-to-bipolar transition leading to their stagnation in the multipolar phase or accumulation in the IZ, and reduced success in populating the CP. In essence, we posit that neuronal polarization is fundamentally distorted in the absence of BAF complex. In support of this notion, we found downregulation of cytoskeleton-related factors in the BAF complex-deleted cortex. More so, adhesion proteins like α-catenin, which orchestrate the plastic linkage between the cell membrane and the internal cytoskeleton to afford cell (neuronal) structure remodeling (Drees et al., 2005; Desai et al., 2013), is lost in response to BAF complex inactivation in the developing cortex. Our investigation of Golgi apparatus localization, which can also indicate alteration in cell polarization, corroborates aberrant neuronal polarization and resultant disturbance of multipolar-to-bipolar morphology transition consequent to BAF complex deletion. A common outcome of improper neuronal polarization during radial migration is the disturbance of axon and dendrite formation, which in the case of the latter, we observed as truncation of the leading process (future dendrite) of the BAF complex mutant migrating neurons. Additionally, improperly polarized radially migrating neurons lacking BAF155 and BAF170 may overly adopt the non-pia surface-directed mode of radial migration, i.e., multipolar migration (Tabata and Nakajima, 2003), which can be a reason for the observed spreading of neurons and cortical mislamination. Future studies seeking to determine how the BAF complex regulates neuronal process elaboration and extension will enrich the literature on how chromatin remodelers orchestrate cortical circuitry.

Together, our investigations show that the BAF complex modulates contact guidance necessary for radial neuronal migration and cortical laminar formation. Key among them is that the BAF complex is essential for maintenance of RG fiber scaffolds, cell adhesion, and neuronal polarization that are indispensable for locomotion of cortical neurons.

### BAF Complex May Suppress WNT Signaling to Drive Cortical Neuron Migration

Beyond the mechanistic cellular intricacies involved in the regulation of cortical neuron migration by the ATP-dependent chromatin remodeling BAF complex, we were interested in identifying a unified molecular mechanism through which BAF complex acts to control radial neuronal migration and cortical laminar formation in the developing brain. We started by screening for possible molecular candidates imputable to our BAF complex mutation-induced neuronal migration phenotype. The WNT signaling pathway emerged as the most plausible candidate among a complex mix of factors altered in the mutant cortex. Unlike other identified signaling pathways (e.g., BMP, SHH, Notch [data not shown]) altered in the BAF complex mutant cortex, WNT signaling appeared consistently elevated in all three dcKO models employed in the study. Moreover, the disturbance of WNT signaling and its effectors are known to cause abnormal neuronal migration (Poschl et al., 2013; Boitard et al., 2015; Bocchi et al., 2017). Admittedly, while WNT activity is markedly increased in the dcKO_Emx1-Cre and dcKO_hFGAP-Cre cortex [see RNA-seq data sheets in Narayanan et al. (2015) and Nguyen et al. (2018), respectively], the dcKO_Nex-Cre cortex displayed mild elevation in WNT signaling probably due to alteration in indirect WNT signaling effectors, including Rspo2 (Lebensohn and Rohatgi, 2018). Perhaps, the perturbation in WNT signaling in the dcKO_Nex-Cre cortex may be mild enough to allow some degree of normal cortical layer formation at later postnatal stages of cortical development, which may not be in the case of the dcKO_Emx1-Cre or dcKO_hFGAP-Cre cortex.

By downregulating the increased WNT signaling activity in the BAF mutant (dcKO_hFGAP-Cre) cortex, we were able to substantially obviate abnormal radial migration of neurons and cortical layer malformation. The neuronal migration rescue upon WNT signaling knockdown is partly due to the preservation of glial fibers, cell adhesion, and cell polarization. Thus, our rescue experiment showed that the BAF complex likely modulates WNT signaling to allow optimal establishment of the requisite molecular and cellular conditions for normal oriented neuronal migration and proper patterning of neocortical layers (Figure 6). A possible explanation for the WNT inhibition-mediated rescue of the migration phenotype is that BAF complex may suppress WNT signaling in neurons en route to their laminae in the CP. In support of our hypothesis, it was previously shown that BAF complex can inhibit WNT/β-catenin signaling via its subunit BAF250b (Vasileiou et al., 2015). It was also reported that WNT signaling is dynamically regulated to allow correct neuronal polarity formation and neuron-glial fiber engagement during radial migration (Boitard et al., 2015; Bocchi et al., 2017). When WNT signaling was ablated in the developing cortex, it resulted in delay of radial migration leading to cortical malformation (Boitard et al., 2015; Bocchi et al., 2017), as phenocopied in the BAF complex mutant (dcKO_Nex-Cre) cortex. We demonstrated in our previous work that BAF complex possibly modulates WNT signaling to permit the establishment of epigenetic schemes required for proper neuronal development during corticogenesis (Nguyen et al., 2018). WNT signaling is indeed a formidable regulator of cortical development as it is reported to regulate many aspects of brain development, including primary forebrain patterning, RG and neuronal precursor cell fate, cell adhesion and polarity formation, and cortical laminar patterning (reviewed in Harrison-Uy and Pleasure, 2012). Nonetheless, due to the partial rescue of the migration phenotype by WNT inhibition, we think other (signaling) factors may be involved. For instance, it could be that WNT inhibition also leads to some normalization of the other dysregulated signaling pathways in the developing BAF complex mutant cortex. As a future consideration, it would be interesting to elucidate the epiphenomenal aspect of our rescue experiment to consolidate our findings.

Altogether, this current study highlights the indispensability of the ATP-dependent chromatin remodeling BAF complex in the formation of cortical layers through regulating multiple aspects of radial neuronal migration in a WNT signaling dependent manner during mammalian cortical development.

## Data Availability Statement

The original contributions presented in the study are publicly available. These data can be found here: https://www.ncbi.nlm.nih.gov/geo/query/acc.cgi?acc=GSE175362.

## Ethics Statement

The animal study was reviewed and approved by the Animal Welfare Committees of the University Medical Center Göttingen and local authority (LAVES: Niedersächsisches Landesamt für Verbraucherschutz und Lebensmittelsicherheit) under the license numbers: 14/1636 and 16/2330.

## Author Contributions

GS performed most of the phenotype characterization and all statistical analyses. CK generated RNA-seq data. HN, LP, and JR contributed to histological analyses. RW did FISH experiment and provided data in Figure 2A. HPN, AF, and JS provided research resources and contributed to discussions. GS and TT wrote the manuscript. TT conceived and supervised the work. All the authors contributed to the article and approved the submitted version.

## Conflict of Interest

The authors declare that the research was conducted in the absence of any commercial or financial relationships that could be construed as a potential conflict of interest.
