# Supplementary material for: Loss of BAF Complex in Developing Cortex Perturbs Radial Neuronal Migration in a WNT Signaling-Dependent Manner
Source: Front Mol Neurosci. 2021 Jun 16;14:687581. doi: 10.3389/fnmol.2021.687581 (PMC8243374; doi:10.3389/fnmol.2021.687581)
Supplement: Supplementary file 5 [file Table_5.docx]

**Supplemental data**

**Figure S1**

**
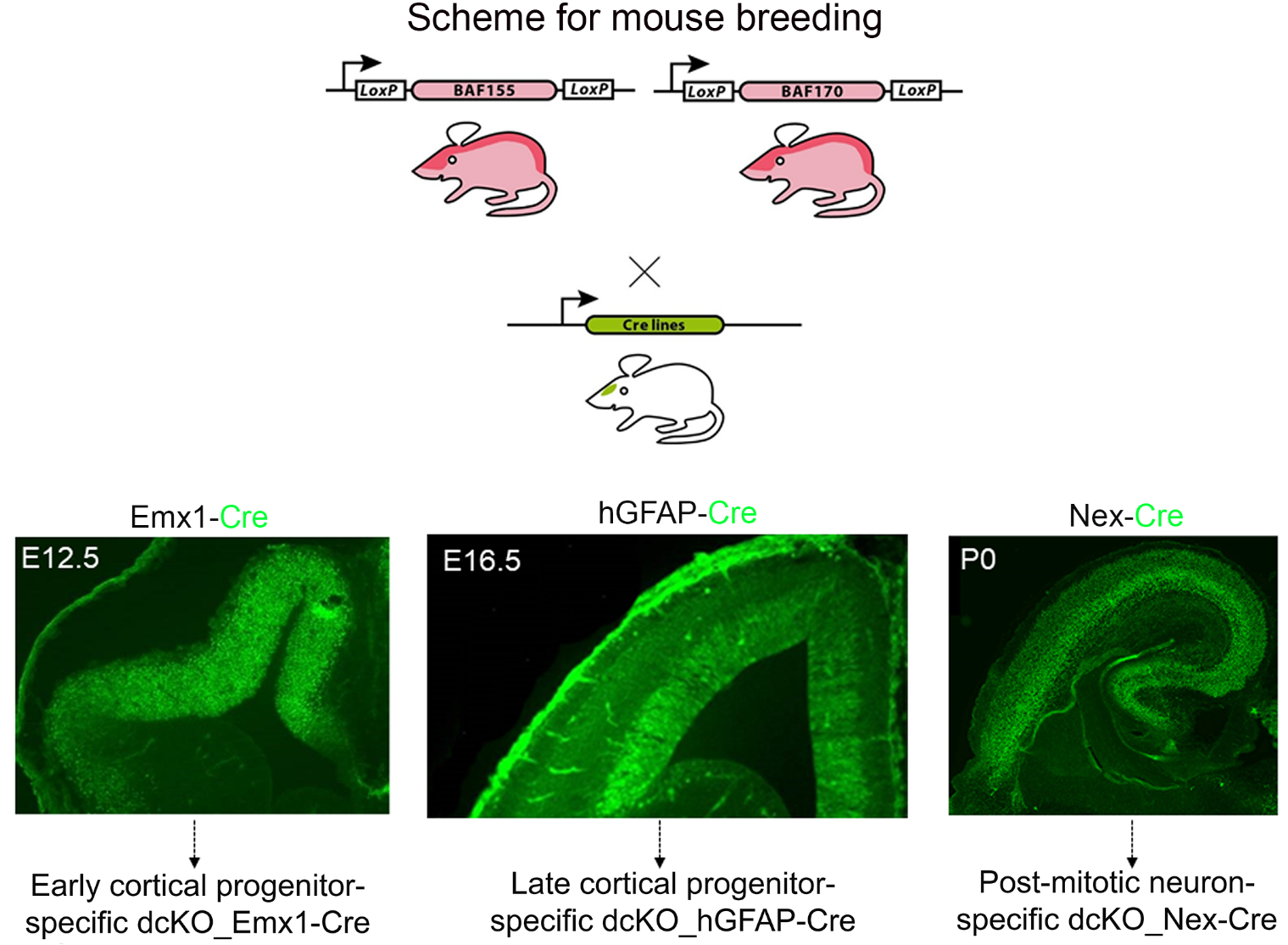
**

**Figure S1.** **Generation of BAF155 and BAF170 knockout mouse lines**. Schema showing the generation of the double conditional knockout (dcKO) mice used in the study, and the corresponding immunohistochemical micrographs of the cortex indicating Cre recombinase activity at the specified cortical development stages.

**Figure S2**


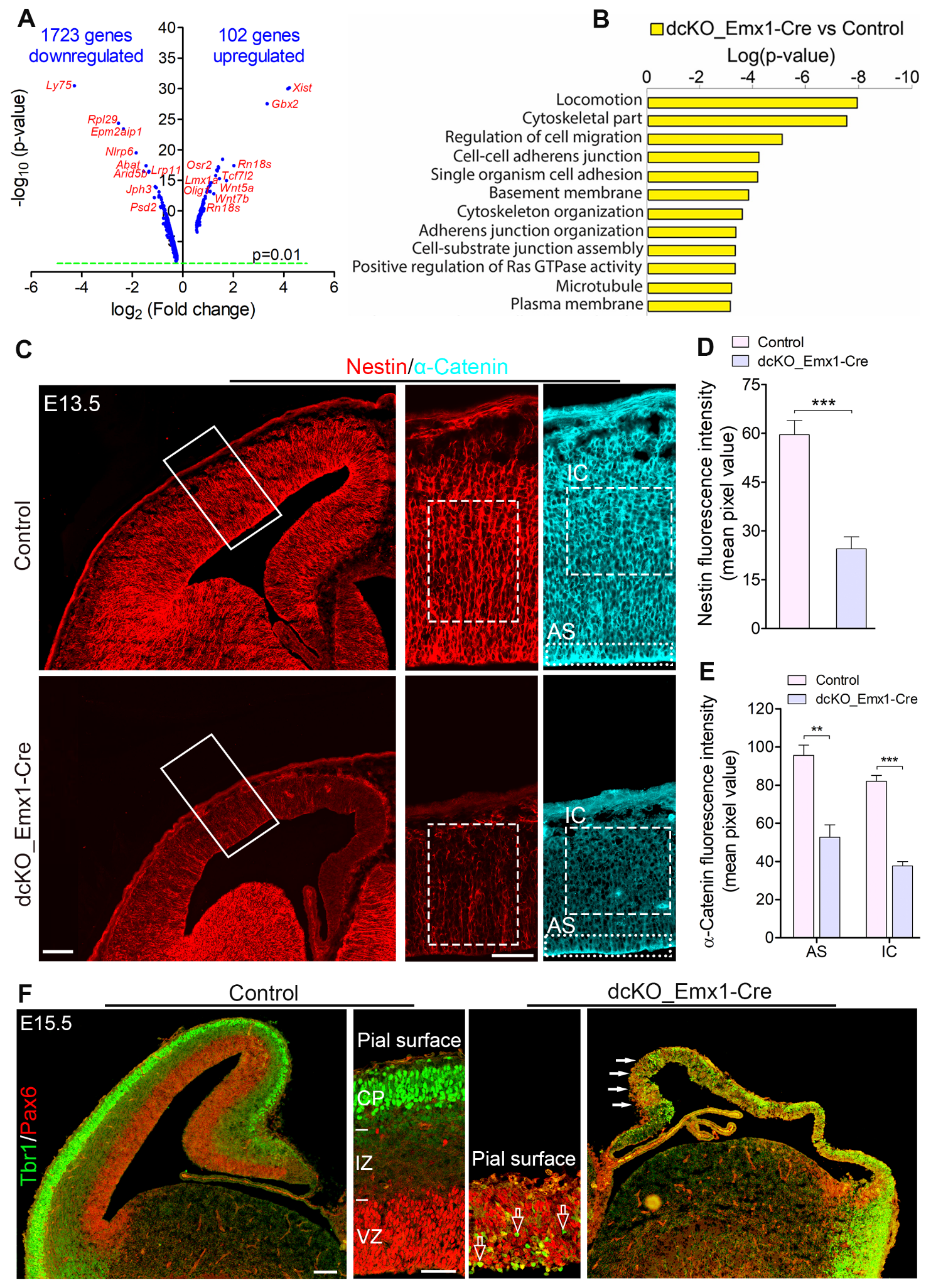


Figure S2. BAF complex-deficient early developing cortex lacks critical neuronal migration factors and appears severely malformed. (A) Volcano plot showing genes downregulated and upregulated in the E12.5 dcKO_Emx1-Cre cortex. Examples of topmost differentially expressed genes are indicated. The total number of genes downregulated and upregulated in the E12.5 dcKO_Emx1-Cre cortex as compared with control are indicated. (B) Graphical representation of gene ontology analysis of RNA-seq results for the E12.5 dcKO_Emx1-Cre cortex versus (vs) control showing downregulation of migration-related gene categories. (C) Sections of E13.5 control and dcKO_Emx1-Cre cortex immunostained for the glial fiber protein Nestin and the cell adhesion protein α-Catenin. White rectangular insets indicate selected cortical areas for quantifications. Specifically quantified cortical areas are shown with rectangles with dashed or stippled lines. (D, E) Simple (D) and grouped (E) bar charts showing quantification of Nestin and α-Catenin expression, respectively, in the E13.5 control and dcKO_Emx1-Cre cortex. (F) Immunohistochemical staining of E15.5 coronal sections of the control and dcKO_Emx1-Cre mouse cortex with antibodies against the early-born layer 6 neuronal marker Tbr1, and the neural stem/progenitor cell marker Pax6. White solid arrows point to the mutant medial cortex with most severe neuronal migration defect. Empty arrows indicate interspersion of non-migrated Tbr1+ L6 neurons with Pax6+ progenitors. Where shown, sections are counterstained with DAPI (blue). Unpaired Student’s *t*-test was used to test for statistical significance: **p < 0.001, ***p < 0.0001; n = 4. Scale bars: = 100 µm and 50 µm in overview and zoomed images, respectively. Results are presented as mean ± SEM. Abbreviations: VZ, Ventricular zone; IZ, Intermediate zone; CP, Cortical plate; IC, Intracortical; AS, Apical surface. The reanalyzed raw RNA-seq data generated from the E12.5 control and dcKO_Emx1-Cre cortex were obtained from previously published work (Narayanan et al., 2015).

**Figure S3**


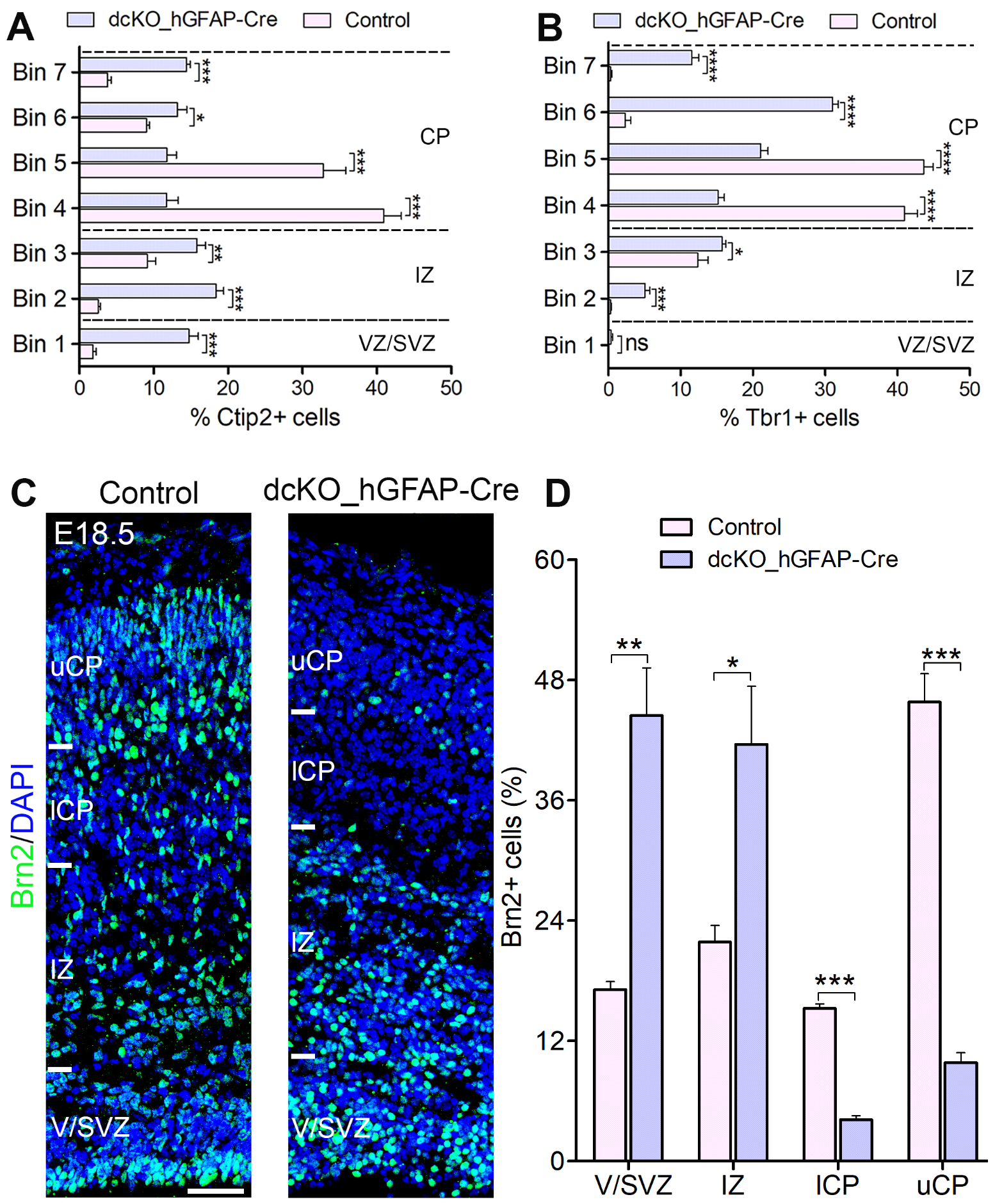


**Figure S3: Neurons are mis-distributed in the perinatal developing cortex lacking BAF complex.** (A, B) Bar charts showing quantitative distributions of deep layer neurons [Ctip2+ (A) and Tbr1+ (B)] in the P0 control and dcKO_hGFAP-Cre cortical wall in Fig 1I. (C) Micrographs showing Brn2 immunostaining in the E18.5 control and dcKO_hGFAP-Cre cortex. DAPI counterstaining is shown. (D) Bar chart comparing the distribution of Brn2+ neurons in the E18.5 control and dcKO_hGFAP-Cre cortical wall. Quantified cortical area = (530 µm x 160 µm^2^). Unpaired Student’s *t*-test was used to test for statistical significance: *p < 0.05, **p < 0.005, ***p < 0.0005, ****p < 0.00005; ns, not significant; n = 6. Scale bar = 50 µm. Abbreviations: VZ, Ventricular zone; SVZ, Subventricular zone; IZ, Intermediate zone; (u/l) CP, (upper/lower) Cortical plate.

**Figure S4**


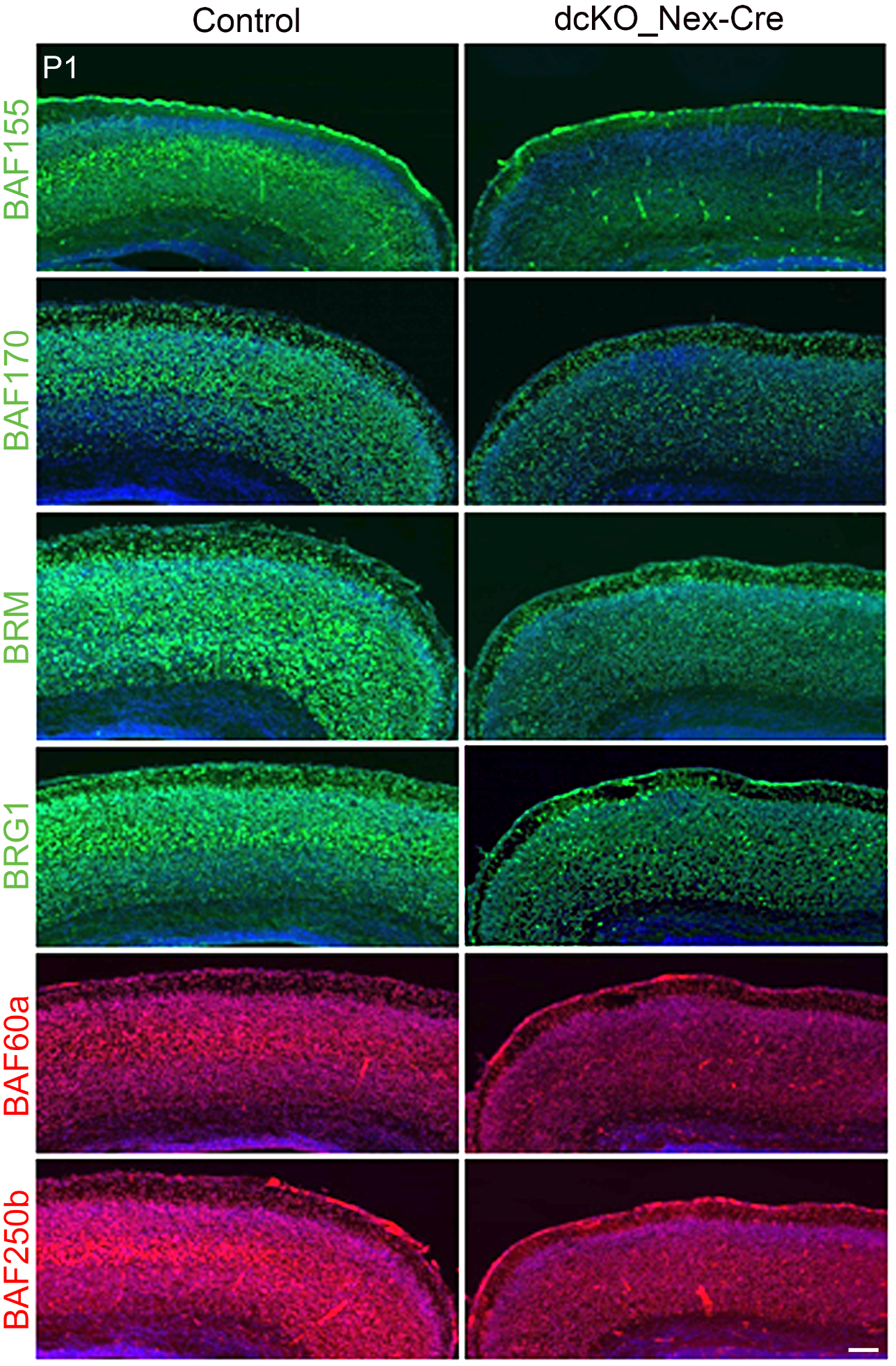


Figure S4: Loss of BAF complex subunits in cortical neurons due to BAF155 and BAF170 deletion. Micrographs showing immunostaining of the various indicated (core) BAF complex subunits in the P1 control cortex and relative depleted levels in the dcKO_Nex-Cre cortex. Scale bar = 100 µm.

**Figure S5**


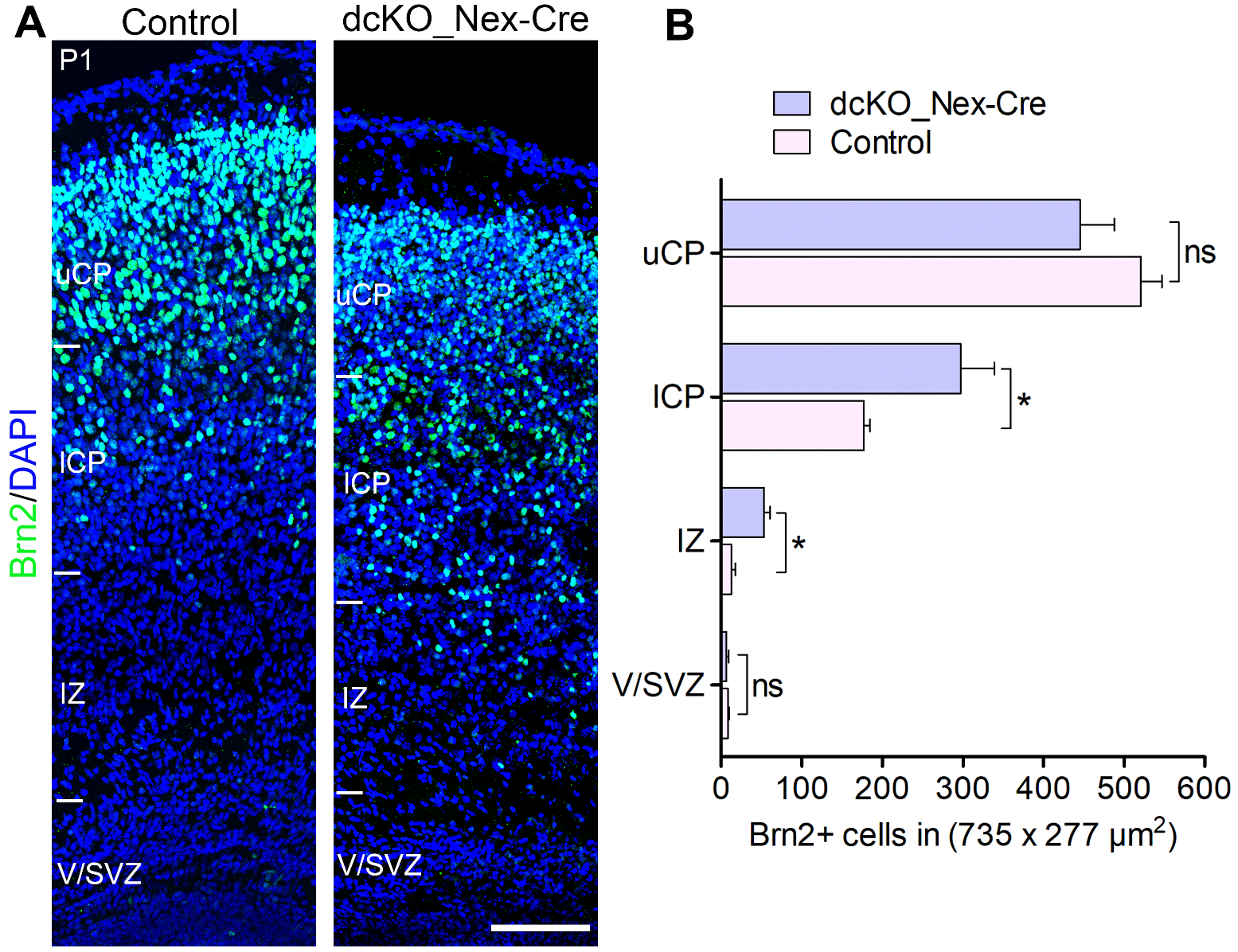


**Figure S5: BAF complex-deficient upper layer neurons expressing Brn2 show mild defect in radial migration.** (A) Micrographs showing Brn2 immunostaining in the P1 control and dcKO_Nex-Cre cortex. DAPI counterstaining is shown. (B) Bar chart comparing the distribution of Brn2+ neurons in the P1 control and dcKO_Nex-Cre cortical wall. Quantified cortical area = (735 µm x 277 µm^2^). Unpaired Student’s *t*-test was used to test for statistical significance: *p < 0.05, ns, not significant; n = 3. Scale bar = 100 µm. Abbreviations: VZ, Ventricular zone; SVZ, Subventricular zone; IZ, Intermediate zone; (u/l) CP, (upper/lower) Cortical plate.

**Figure S6**

**­­
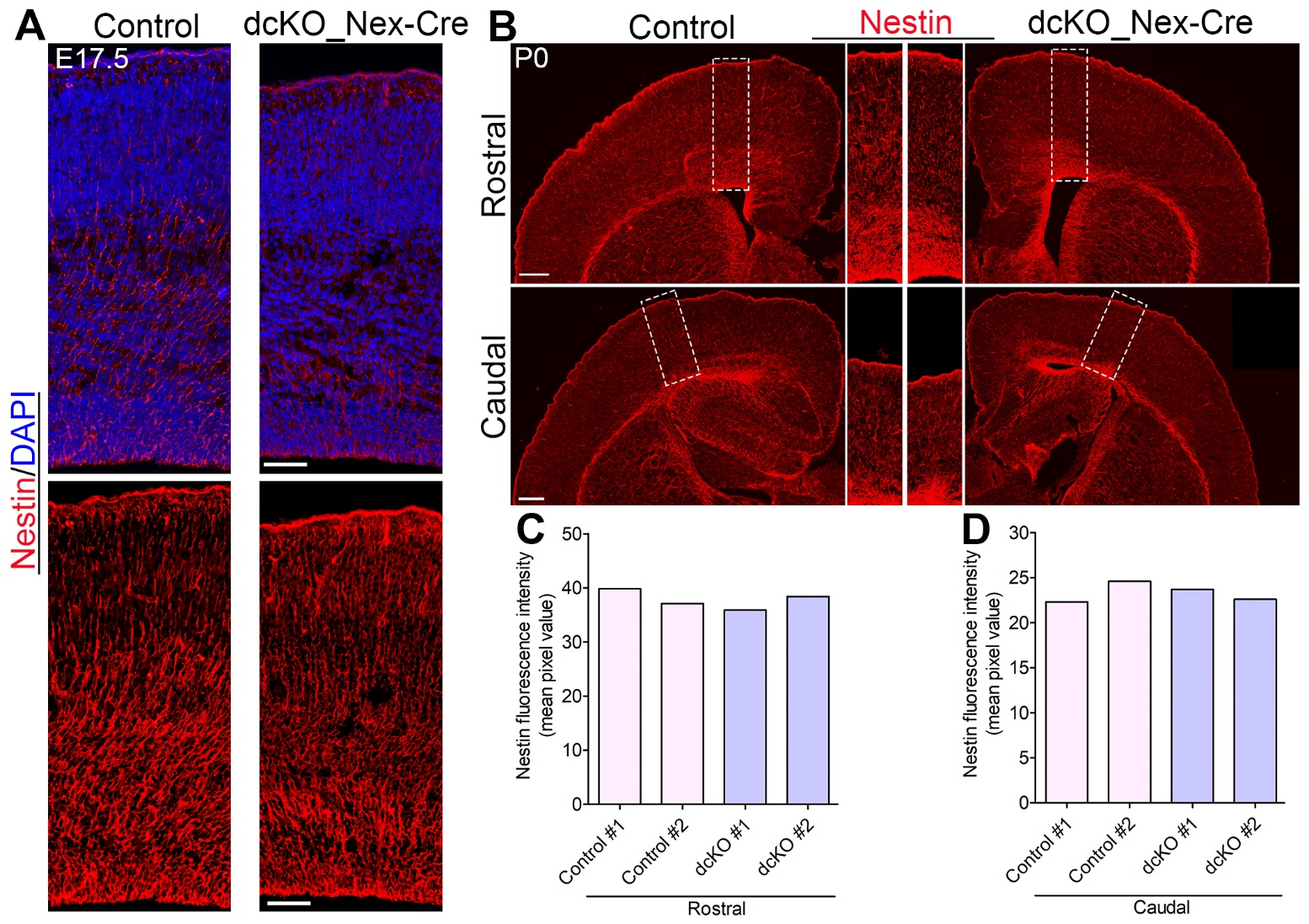
**

**Figure S6:** **Radial glial fibers are not disturbed when BAF complex is ablated in postmitotic neurons in the developing cortex.** (A) Immunomicrographs showing comparable Nestin staining in the E17.5 control and dcKO_Nex-Cre cortex. DAPI counterstaining is shown. (B) Immunomicrographs showing Nestin staining in the rostral and caudal aspects of the P0 control and dcKO_Nex-Cre cortex. Cortical areas selected for quantification are indicated. (C, D) Simple bar graphs showing comparable density of radial glial fibers in the rostral (B) and caudal (C) aspects of the P0 control and dcKO_Nex-Cre cortex. n = 2. Scale bar = 50 µm (A), 200 µm (B).

**Figure S7**


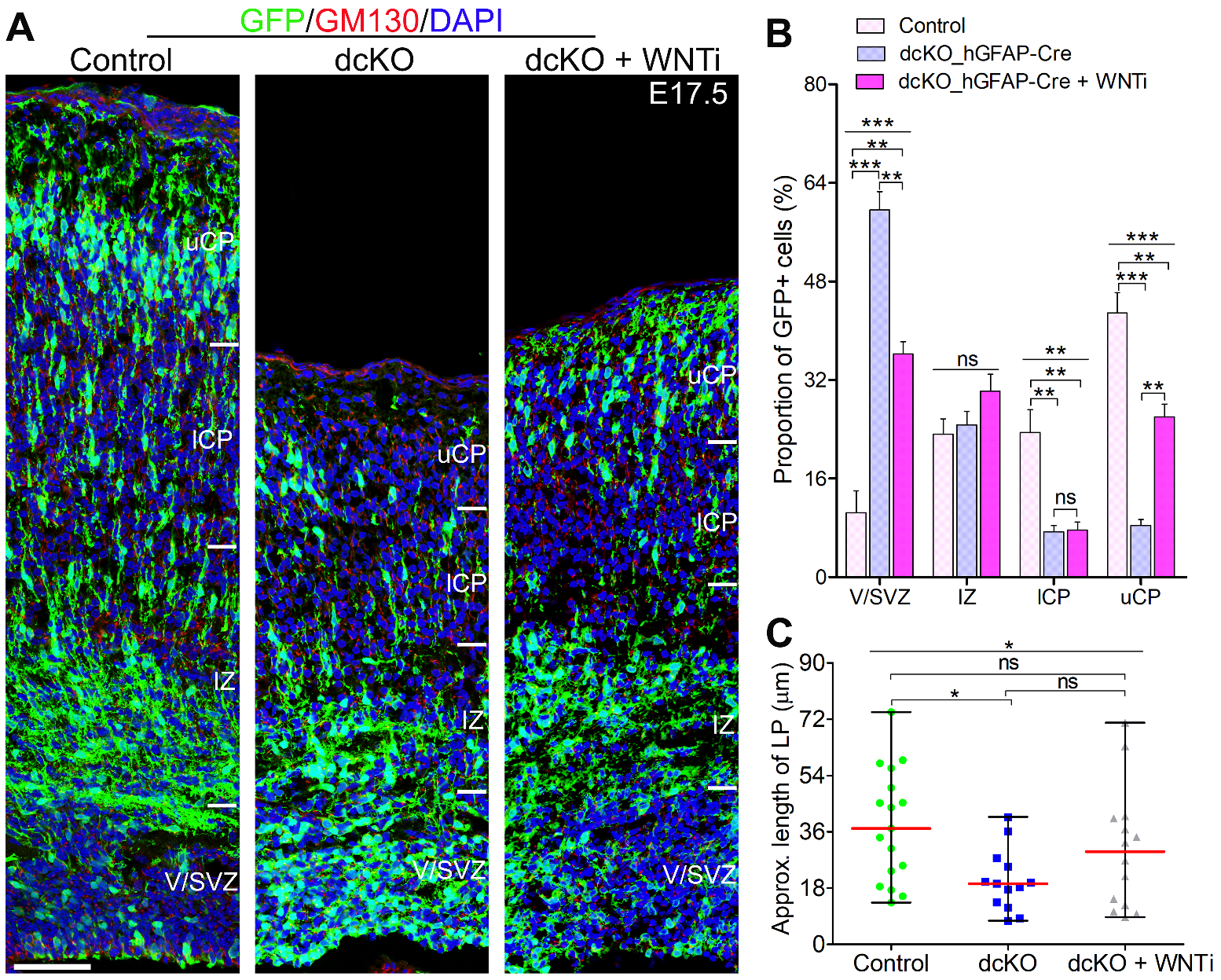


**Figure S7:** **WNT inhibition rescues neuronal migration defect in the BAF complex mutant cortex.** (A) Immunomicrographs showing migrating neurons labeled with GFP and GM130 in the E17.5 control, dcKO_hGFAP-Cre without WNT inhibitor treatment, and dcKO_hGFAP-Cre with WNT inhibitor treatment cortex. In utero electroporation was done at E14.5. DAPI counterstaining is shown. (B) Bar graph showing the distribution of migrating GFP-labeled neurons in the cortical wall of 3 sets (n = 3) of cortices as shown in (A). (C) Graph comparing the median estimated leading process length of control neurons and neurons treated with or without WNT inhibitor in the lower cortical plate. *p < 0.05, **p < 0.005, ***p < 0.0005, ns, not significant. Scale bar = 50 µm. Abbreviations: V/SVZ, Ventricular/Subventricular zone; IZ, Intermediate zone; (u/l) CP, (upper/lower) Cortical plate.
